# Long-Term Safety and Efficacy of CD19 Humanized Selective CAR-T Therapy in B-ALL Patients Who Have Previously Received Murine-Based CD19 CAR-T Therapy

**DOI:** 10.3389/fonc.2022.884782

**Published:** 2022-06-21

**Authors:** Yu Zhao, Jianping Zhang, Junfang Yang, Huantong Wu, Yao Chen, Nannan Li, Zhongfeng Liu, Xuan Wang, Weihua Liu, Guangji Zhang, Bin-Bing Stephen Zhou, Peihua Lu, Zhiguo Chen

**Affiliations:** ^1^ Cell Therapy Center, National Clinical Research Center for Geriatric Diseases, and Key Laboratory of Neurodegenerative Diseases, Ministry of Education, Beijing Institute of Geriatrics, Xuanwu Hospital Capital Medical University, Beijing, China; ^2^ Center of Neural Injury and Repair, Beijing Institute for Brain Disorders, Beijing, China; ^3^ Hebei Yanda Lu Daopei Hospital, Langfang, China; ^4^ Beijing Lu Daopei Institute of Hematology, Beijing Lu Daopei Hospital, Beijing, China; ^5^ Key Laboratory of Pediatric Hematology and Oncology Ministry of Health, Pediatric Translational Medicine Institute, Department of Hematology and Oncology, Shanghai Children’s Medical Center, Shanghai Jiao Tong University School of Medicine, Shanghai, China

**Keywords:** CAR-T, humanized, repeated dosing, selective domain, B-ALL, HSCT

## Abstract

Murine-based CD19 CAR-T (CD19m CAR-T) therapy can lead to a relatively high CR rate when administered to B-ALL patients for the first time. However, the DOR is sub-optimal and a subset of patients even show primary resistance to CD19m CAR-T. To address these issues, we employed a humanized selective CD19CAR-T (CD19hs CAR-T) and evaluated the long-term safety and efficacy of treating 8 R/R B-ALL patients who had relapsed or failed to achieve CR following CD19m CAR-T infusion (Clinical trials’ number: ChiCTR1800014761 and ChiCTR1800017439). Of the 8 patients, 7 achieved CR on Day 30 after the 1^st^ infusion of CD19hs CAR-T. The median CRS grade was 1 without significant neurotoxicity seen in any of the 8 patients. The median DOR was 11 months, significantly longer than the DOR following CD19mCAR-T infusions. Anti-CAR antibodies were induced in patients who had received prior CD19m CAR-T infusions but not in those following a single or repeated CD19hsCAR-T treatment, which probably had contributed to the sub-optimal DOR and/or failure of effective response in these patients. CD19hs CAR-T, in contrast, induced low immunogenicity compared with CD19m CAR-T, suggesting that a repeat dosing strategy might be feasible and efficacious for patients who have relapsed and/or show primary resistance to CD19m CAR-T therapy. In this clinical study, CD19hs CAR-T showed a significant clinical efficacy with mild side effect among patients with R/R B-ALL who had previously received CD19m CAR-T.

**Clinical Trial Registration:**

https://www.chictr.org.cn/showprojen.aspx?proj=25199 (ChiCTR1800014761). https://www.chictr.org.cn/showproj.aspx?proj=29174 (ChiCTR1800017439).

## Introduction

CD19-specific chimeric antigen receptor T-cells (CD19 CAR T-cells) have demonstrated encouraging clinical efficacy for the treatment of relapsed or refractory (R/R) B-cell malignances, including B-cell acute lymphoblastic leukemia (B-ALL), non-Hodgkin lymphoma (NHL) and chronic lymphocytic leukemia (CLL) (1). According to the accumulating evidence, the median event-free survival (EFS) time following CD19 CAR-T treatment among R/R B-ALL, B-NHL and B-CLL patients has been estimated to be 6-12 months (2–4), 3-6 months (5–7), and 3-12 months (8–10), respectively. However, about 50% of patients who had achieved complete remission (CR) relapsed within 1 year, and about 10% -20% of patients failed to respond to a first time treatment of CD19 CAR-T (11–13). Moreover, although it would be a feasible strategy to administer a 2^nd^ infusion to patients who relapsed from or displayed a primary resistance to a 1^st^ infusion of CD19 CAR-T, an objective response was rarely observed, as results from various clinical trials show (11, 12, 14–16). This nonresponse to CAR-T therapy puts these patients into a desperate situation, with no additional therapeutic options. The mechanisms underlying the primary resistance to CAR-T cells and the failure to respond to repeated infusions of this therapy remain elusive. Several possible mechanisms proposed include the loss of tumor target expression (negative relapse), poor T-cell function and insufficient persistence of the engineered T-cells (positive relapse), an immunological rejection (positive relapse), and an immunosuppressive tumor microenvironment (11, 12, 14, 15, 17, 18). Previous reports from our lab and those of others have shown that CD19 CAR-T with murine-based scFv can be blunted and/or rejected by anti-CAR specific immune response during repeat infusions or even a first CAR-T infusion (16, 19).

To overcome these obstacles associated with murine-based CD19 CAR-T (CD19m CAR-T) therapy, humanization of CD19 CAR has been attempted by several groups to improve the clinical response of CD19 CAR-T therapy. The results reported by these groups suggested that humanized CD19 CAR-T can achieve significant therapeutic efficacy in the treatment of patients with R/R B cell malignancies (20–23). Our group also developed a humanized CD19 CAR with a high binding affinity to CD19. The CD19CAR includes a selective domain between the heavy and light chains of scFv rendering it selectively expandable *via* treatment with the selective domain-specific monoclonal antibodies (16, 19). In this phase I clinical trial, we evaluated the long-term safety and efficacy of the humanized selective CD19 CAR-T (CD19hs CAR-T) in treating 8 patients who had relapsed from or shown primary resistance to CD19m CAR-T therapy. The results from our small 8 patient cohort demonstrate that CD19hs CAR-T exerted a significant anti-tumor effect with very mild side effects. Importantly, the anti-tumor efficacy was still observed after repeated infusions of CD19hs CAR-T.

## Methods and Materials

### Study Design and Patient Enrollment

The phase I clinical trial (ChiCTR1800014761 and ChiCTR1800017439) aimed to evaluate the safety and efficacy of CD19hs CAR-T treatment. Major inclusion criteria were: (1) Age < 75 years; (2) CD19+ relapsed/refractory B-cell lymphoma, acute B lymphocytic leukemia, or chronic B lymphocytic leukemia, including patients who had previously received murine-based CD19CAR-T infusion(s); and (3) MRD-positive relapsed/refractory acute B lymphoblastic leukemia, B cell lymphoma, or chronic B lymphoblastic leukemia. The protocols were approved by the Ethics Committee of Xuanwu Hospital Capital Medical University, and the Ethics Committee of Hebei Yanda Lu Daopei Hospital. All patients enrolled and treated in this trial signed a written informed consent before participation. All clinical investigations were conducted according to the Declaration of Helsinki principles.

### CD19hs CAR-T Production

CD19hs CAR-T cells were produced as previously described (16, 19). Briefly, PBMCs from the patients or donors were collected by leukapheresis. CD3-positive T lymphocytes were enriched and activated with CD3/CD28 magnetic beads. Activated T-cells were transduced with lentiviral vectors expressing CD19hs CAR, and were stimulated with a monoclonal antibody specific to the selection domain (SmAb) inserted between the heavy and light chains of scFv, during the expansion stage *in vitro*. CD19hs CAR-T cells were harvested and cryopreserved after a 10-day culture. The final product was released for clinical administration after passing a quality control test according to a previous description (16). The detailed information of final products is listed in **Table S5**.

### Quantitative Analysis of CD19hs CAR-T Cells After Infusion

Flow cytometry was used to analyze the percentage of CD19hs CAR-T cells in PB and BM samples with a biotin-labeled Protein-L (ACROBiosystems, USA) and PE-Streptavidin antibody (Biolegend, USA). CD3-positive T cells were stained by using mouse anti-human CD3-FITC monoclonal antibody (Biolegend, USA). CD19hsCAR copy numbers were quantified by using qPCR as previously described (24).

### Measurement of Cytokine Levels

Serum levels of sCD25 (soluble IL-2 receptor), interleukin-6 (IL-6), interleukin-10 (IL-10), and interferon-γ (IFN-γ) were analyzed using ELISA or an electrochemiluminescence (MSD) assay (Meso scale discovery, MD, US) following the manufacturer’s instructions. ELISA data were acquired with VARIOSKAN FLASH (Thermo Scientific), and MSD assay data were acquired with the QuickPlex SQ120 system (Meso Scale Diagnostics).

### Detection of Anti-CAR Immunoglobulins

CAR-specific immunoglobulins, including IgA, IgG and IgM were measured as previously described (16). In brief, sera were collected from the patients before and after CD19hs CAR-T infusion. The recombinant extracellular domain of CD19m CAR and CD19hsCAR (1 mg/mL in PBS) were diluted to 4 μg/mL using 0.1 mmol/L PBS, and were coated to the bottom of 96-well ELISA plates. Test wells were blocked for 30 min at 37°C using 1% BSA. Samples (100 μL) were added into the wells and incubated for 1 h at 37°C. HRP-labeled goat-anti human antibodies specific for IgA, IgG, or IgM (Beijing Zhuang-Meng Biotechnology Co., Ltd) were added into the wells after 5 washes. TMB substrate was used for color development, and measurement was performed by using microplate reader at 450 nm. Positive results were set at an OD_450_ value >=0.2.

### Statistical Analysis

Data were presented as a median with a range, mean with range, or mean ± SEM depending on the analysis settings. Statistical analysis was conducted using Prism Software (GraphPad Software, CA, US). For comparison between two groups, Student’s t-test was conducted as a two-sided paired test with a confidence interval of 95%. For comparisons of three groups or more, the analysis was performed by using one-way ANOVA with Dunnett’s multiple comparisons test. Results with a p-value less than or equal to 0.05 were considered significant.

## Results

### Patients and Disease Characteristics Prior to CD19hs CAR-T Treatment

**Figure 1:**
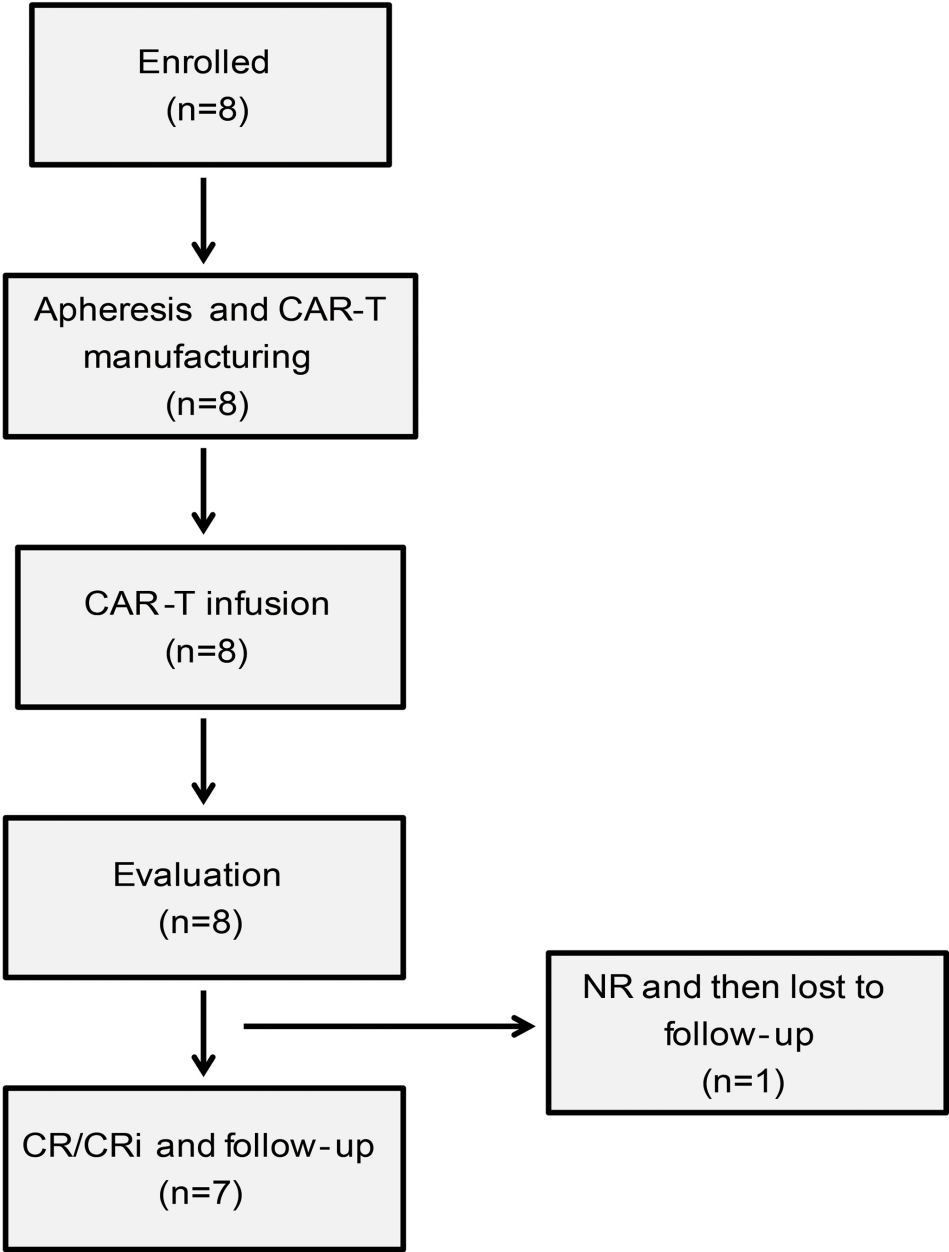
The flowchart of CD19hsCAR-T treatment.

Eight R/R B-ALL patients were enrolled in the clinical trials (www.chictr.org.cn, ChiCTR1800014761 and ChiCTR1800017439) with written informed consent (**Figure 1**). The eligibility for enrollment included subjects with B-ALL, B-NHL and B-CLL; yet the actual recruitment was limited to B-ALL due to availability issues. The general information of patient characteristics is summarized in **Table 1** and **Table S1**. All of the patients included in this trial were heavily pre-treated. Six of the 8 patients (No.1-6) were diagnosed with fusion gene-positive disease, including E2A-HLF (No.1 and 2), BCR-ABL1 (No.3 and 6) and MLL/ITD (No.4 and 5). Three of the 8 patients (No.2, 5 and 6) were diagnosed with complex chromosomes. The median number of previous therapeutic regimens was 6 (range, 3-8). Prior to CD19hs CAR-T infusion, all of the 8 patients had received CD19m CAR-T treatment at least once. Of these 8 patients, 1 patient (No.4) received a single dose of CD19m CAR-T. Three patients (No.3, 5 and 7) received 2 repeated infusions of CD19m CAR-T. Three patients (No.1, 2 and 6) received a combination of CD19m CAR-T and CD22m CAR-T, with 2 of the 3 patients (No.1 and 6) received the infusion in the primary administration, and 1 of the 3 (No.2) received the infusion upon relapse of a CD19m CAR-T-induced CR. In particular, of the 8 patients, 1 patient (No.8) received 1 infusion of humanized CD19 CAR-T (from a different hospital) after showing a non-response (NR) to CD19m CAR-T. Four patients (No.4, 5, 6, and 8) were subjected to a bridging to allo-HSCT regimen after achieving a CR with a CD19m CAR-T infusion. One patient (No.3) who had central nervous system involvement received allo-HSCT before the first CD19m CAR-T treatment (**Table S1**). Four patients (No.1, 2, 3 and 7) were not subjected to the allo-HSCT bridging regimen following CD19m CAR-T treatment. For these 4 patients, the median duration of remission (DOR) was 2.25 months (range, 0-8) after the 1^st^ CD19m CAR-T infusion. In contrast, the median DOR was much longer, 11.5 months (n=4, range, 6-18) for the patients who were subjected to the bridging to HSCT regimen after the 1^st^ CD19m CAR-T treatment (**Figure 6D**). Furthermore, 3 patients (No.2, 3 and 7) received a second infusion of CD19m CAR-T, with one patient relapsing within 1 month, and 2 patients showing nonresponse to CD19m CAR-T. Primary resistance to CD19m CAR-T was observed in 3 patients (No.5, 7 and 8). Patient No.7 failed to achieve CR after 2 repeat CAR-T infusions. Whereas patient No.5 achieved CR after the second infusion of CD19m CAR-T, and continued with the bridging to allo-HSCT regimen, patient No.8 patient achieved CR following treatment with a humanized CD19 CAR-T (from a different hospital), and continued with the bridging to allo-HSCT regimen.

**Table 1:** Patients’ disease characteristics prior to CD19hs CAR-T infusion.

Patient No.	Age	Sex	Complex chromosome	Gene fusion	Previous CAR-T therapies				Outcomes and DOR (mon) after mCAR-T infusions	Bridging to HSCT	No. of treatment regimens before enrollment
					Source of CAR scFv	Target	No. of infusions	Infusion dosage×10^6/kg			
1	9	M	N	E2A-HLF	murine	CD19 + CD22	1	0.3 + 0.3	CR with MDR- for 1	N	6
2	14	M	Y	E2A-HLF	murine	CD19	2	0.3	CR with MRD- for 3.5	N	3
						CD19 + CD22		0.3 + 0.3	NR		
3	17	M	N	BCR-ABL1	murine	CD19	2	4	CR with MRD- for 8	N	6
						CD19		0.3	CR with MRD- for 1^*^		
4^$^	14	F	N	MLL/ITD	murine	CD19	1	1	CR for 18^**^	Y	4
5^$$^	20	F	Y	MLL/ITD	murine	CD19	2	1	NR	Y	6
						CD19		1	CR with MRD- for 12^***^		
6^$$$^	6	F	Y	BCR-ABL1	murine	CD19 + CD22	1	0.9 + 1	CR with MRD- for 11	Y	6
7^$$$$^	19	M	N	N	murine	CD19	2	0.06	NR	N	8
						CD19		0.5	NR		
8	13	F	N	N	murine	CD19	2	0.06	NR	Y	6
					hu	CD19		0.64	CR for 6^****^		

allo-HSCT, allogeneic hematopoietic stem cell transplantation; CD19hsCAR-T, chimeric antigen receptor T cells engineered with humanized selective CD19-specific scFv; mCAR-T, chimeric antigen receptor T cells engineered with murine-based scFv; mon, month(s); CR, complete remission; DOR, duration of remission, time spanning from CR as evaluated on day 30 post-infusion to either the time of relapse, death, loss to follow-up, or the present time when the manuscript was prepared in the case of ongoing sustained CR; MRD, minimal residual disease; hu, humanized; ITD: internal tandem duplication; F, female; M, male; N, no; NR, nonresponse; Y, yes.

^$^Patient 4 harbored gene mutations, including IKZF1 mutation, ERG (Δ3-9 positive), FANCD2 (C2080 G>A pD694N), NRAS (G13D) and JAK (I668F);

^$$^Patient 5 harbored gene mutations, including IKZF1 mutation, ERG (Δ3-9 positive) and NRAS (G13D);

^$$$^Patient 6 harbored gene mutation, including IKZF1 heterozygous deletion from Exo5-6, PAX5 heterozygous deletion from Exo 2-6, and Exo 8;

^$$$$^Patient 7 harbored gene mutation, including KRAS Q22K and ASXL1 T822Pfs*3;

*Patient 3 received the 2^nd^ infusion of CD19mCAR-T as a preventive treatment;

**Patient 4 achieved CR for 18 mon with CD19mCAR-T infusion bridging to allo-HSCT;

***Patient 5 achieved CR for 12 mon with 2 consecutive CD19mCAR-T infusions, then bridging to allo-HSCT;

****Patient 8 did not respond to CD19mCAR-T, and then achieved CR for 6 mon with a humanized CD19CAR-T (from a different research group; CR with 0.11% MDR) bridging to haplo-HSCT (CR with MRD-).

### CD19hs CAR-T Treatment Regimen

As previously reported (16), all patients were subjected to cyclophosphamide/fludarabine (Cy-Flu) lymphodepletion preconditioning prior to CD19m CAR-T and CD19hs CAR-T infusions (cyclophosphamide, 250 mg/m^2^ for 3 days; fludarabine, 30 mg/m^2^ for 3 days). The CD19hs CAR-T dosage was determined by tumor burdens, previous murine CAR-T dosage used, and the response to previous murine CAR-T infusion(s), particularly the severity of cytokines release syndrome (CRS). Two of the 8 patients (No.1 and.2) received autologous CD19hs CAR-T, and 6 of the 8 (No.3-8) were infused with allogeneic CD19hs CAR-T; among the 6, 5 received CAR-T generated from the PBMCs of the same allo-HSCT donors and one received CAR-T produced from HLA fully-matched sibling’s PBMCs. CD19hsCAR-T was administered at a higher dose versus CD19m CAR-T in most patients (6/8), ranging from 0.3×10^6/kg to 3×10^6/kg (details in **Table S6** and **Figure S1**). Of the 8 patients, 2 patients each received a dosage of 1×10^6/kg (No.1 and 3), 2×10^6/kg (No.7 and 8) and 3×10^6/kg (No.4 and 5), respectively. The remaining 2 patients were infused at a dosage of 0.3×10^6/kg (No. 2) and 1.5×10^6/kg (No.6). Patient No. 2 received the same dose of CD19m CAR-T as CD19hs CAR-T, 0.3×10^6/kg (No.2). Patient No. 3 received a reduced dose of 1×10^6/kg CD19hsCAR-T compared to 3×10^6/kg CD19m CAR-T. Patients No.4 and No.5 received the second infusion of CD19hs CAR-T with a dosage of 3×10^6/kg upon relapse following an 11-month CR period following the 1^st^ infusion of CD19hs CAR-T (**Figure S1**).

The median dosage of CD19hs CAR-T was 1.75×10^6/kg for the 1^st^ infusion, approximately 3-fold higher than the counterpart of CD19m CAR-T (0.6×10^6/kg) for the 1^st^ infusion. However, the highest dose of CD19hs CAR-T was lower than that of CD19m CAR-T (3×10^6/kg versus 4×10^6/kg). As to the repeated treatments, a single dose of 3×10^6/kg was administered for CD19hs CAR-T infusion, higher than the dose range of CD19m CAR-T (0.3×10^6/kg to 1×10^6/kg).

### Safety of CD19hs CAR-T

**Figure 2:**
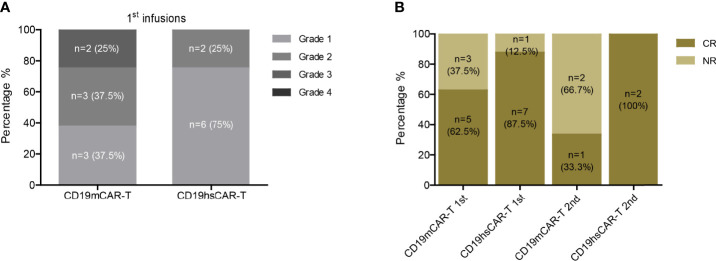
Safety and efficacy of CD19hs CAR-T. **(A)** CRS following CD19m CAR-T and CD19hs CAR-T infusions. **(B)** CR rates after the 1^st^ and 2^nd^ infusions of CD19m CAR-T and CD19hs CAR-T, respectively.

Despite the higher infusion dose of CD19hs CAR-T compared to that of CD19m CAR-T in 75% of patients, we observed low rates of severe cytokine release syndrome (CRS) and neurotoxicity following treatment with CD19hs CAR-T (**Figure 2A** and **Tables S2**, **S3**, **S6**). The median CRS grade after the 1^st^ CD19hs CAR-T infusion was 1 (range, 1-2), and the median CRS grade after the 1^st^ CD19m CAR-T was 2 (range, 1-3, not significantly different, p = 0.1113). Of the 8 patients, 2 patients (No.6 and 8) developed grade 2 CRS after infusion of CD19hs CAR-T. Tocilizumab was administered to manage the CRS. No patients developed neurotoxicity following infusions of CD19hs CAR-T although 1 patient (No.3) had extramedullary relapse in the CNS. This patient displayed grade 1 neurotoxicity after receiving CD19m CAR-T, but not after receiving CD19hs CAR-T. After the 2^nd^ treatment with CD19hs CAR-T, 1 of 2 patients displayed grade 2 CRS and received tocilizumab for CRS management. All of the patients were treated with steroids (5~60 mg per day) to minimize CRS symptoms, with a duration of 2 to 5 days depending on the severity of CRS and the lasting time of fever.

**Figure 3:**
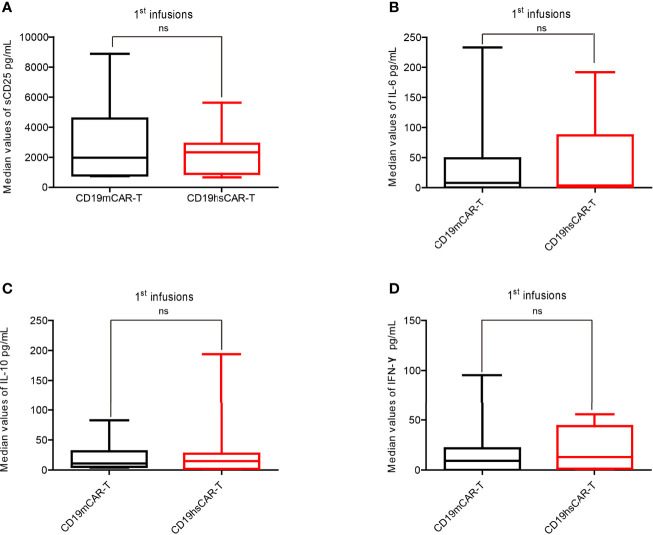
Levels of cytokines in patient sera after the 1^st^ infusion of CD19m CAR-T and the 1^st^ infusion of CD19hs CAR-T. **(A–D)** Comparisons of the median concentration of sCD25 **(A)**, IL-6 **(B)**, IL-10 **(C)** and IFN-γ **(D)**, respectively (n=8). The bars represented the range of concentrations for each cytokines within 30 days after the 1^st^ infusion; medians are shown as straight lines in each bar. P values were determined by T-test, and the significant levels were identified as p<= 0.05. Levels of each cytokine were repeatedly tested 7 times within 30 days after infusion. ns, Not statistically significant.

The levels of various CRS-related cytokines in peripheral blood (PB) were measured and analyzed after CD19hs CAR-T infusion, which included soluble IL-2 receptor (sCD25), IL-6, IL-10 and IFN-γ. Within 30 days after the 1^st^ CD19hs CAR-T infusion, levels of these cytokines all increased, reached peak values, and then tapered off (**Figure S2**). Although the dosages of the 1^st^ CD19hs CAR-T were higher than those of CD19m CAR-T in 75% of the patients, the median values of sCD25, IL-6, IL-10 and IFN-γ in PB were not significantly higher (**Figure 3**). After the 2^nd^ infusions of CD19hs CAR-T, the cytokine response profiles were comparable to those after the 2^nd^ CD19m CAR-T infusions, although CD19hs CAR-T was administered at a higher dosage (**Figure S3**). The results suggest that CD19hs CAR-T is well-tolerated with a considerably safe clinical profile, even when administered at a relatively high dosage.

### Expansion and Persistence of CD19hs CAR-T in Patients

**Figure 4:**
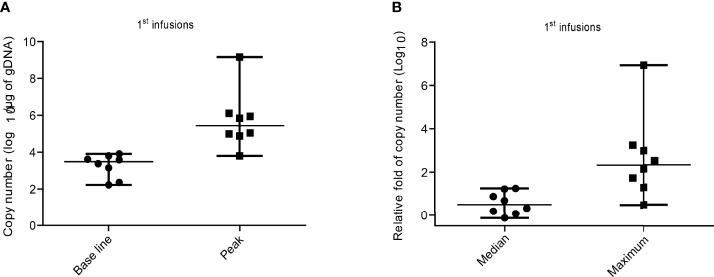
CD19hs CAR-T expansion after the 1^st^ infusion in patients. **(A)** Expansion of CD19hs CAR gene copy numbers (n=8) after infusions. Base line was detected on the Day 0 before CD19hsCAR-T infusion; Peak means the Maximum CAR transgene copy number after infusions. **(B)** The relative fold change of CAR transgene copy numbers after infusions in patients (n = 8). Median means the relative fold medians in patients after 1^st^ infusions; Maximum means the peak values of relative fold in each objects. The data were presented as scatter dots with median and range.

We examined the copy numbers of the CAR transgene by qPCR on blood cells from all patients. As shown in **Figure 4** and **Figures S4A, B** following infusion, we detected a marked expansion of CD19hs CAR-T in all of the patients, but the efficacy was relatively poor in patient No.2 compared with the rest of the subjects (fold change of 2.99). The pre-CD19hs CAR-T Day 0 median of transgene copy number was 3.49log_10_/μg of gDNA (range, 2.23log_10_/μg of gDNA- 3.9log_10_/μg of gDNA). The median of the peak values was 5.6log_10_/μg of gDNA (range, 3.8log_10_/μg of gDNA- 9.2log_10_/μg of gDNA). The median of the relative fold increase at the peak was 2.32Log_10_ (232.5) [range, 0.47log_10_ (2.99)-6.93log_10_ (8.27×10^6)].

**Figure 5:**
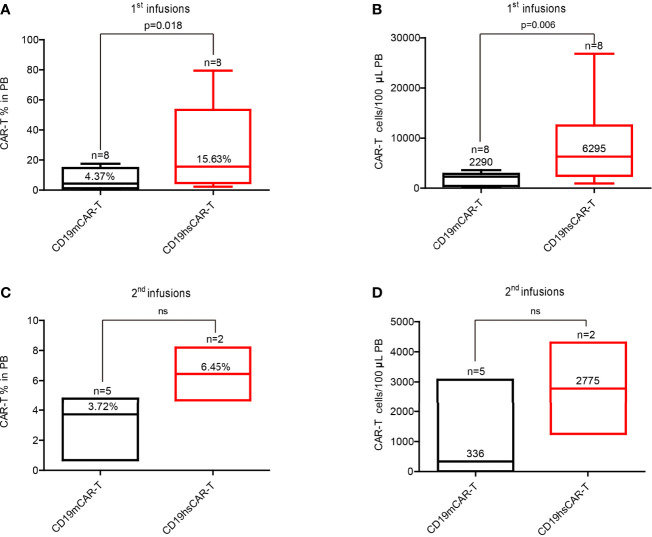
Median of peak values of the CAR-T percentage and cell count in PB after infusion of CD19m CAR-T and CD19hs CAR-T, respectively. **(A, B)** Median of peak values of CAR-T percentage and cell count after the 1^st^ infusions of CD19m CAR-T (n = 8) and CD19hs CAR-T (n = 8). **(C, D)** Median of peak values of CAR-T percentage and cell count after the 2^nd^ infusions of CD19m CAR-T (n = 5) and CD19hs CAR-T (n = 2). Bars represented the range of the peak values within 30 days after infusions. The median values are shown as straight lines in each bar. P values were determined by T-test, and the significant levels were identified as p < = 0.05. Levels of each cytokine were repeatedly tested 7 times within 30 days after infusions. ns, Not statistically significant.

For all patients, the number of CD19hs CAR-T cells was also measured in PB following infusions using flow cytometry (**Figures S4C, D**). Compared with the CD19m CAR T-cells, the peak values of the percentage and/or of the cell count were significantly higher after CD19hs CAR-T infusions in 75% patients (6/8) (**Figures S5**, **S6**). The median of the peak percentage values was 15.63% (range, 2.32%-79.46%), corresponding to a median of peak cell count of 6259 cells/100μL (range, 939 cells/100μL-26800 cells/100μL), which were significantly higher than those associated with CD19m CAR-T treatments (4.38% versus 15.63%, p = 0.018; 2290 cells/100μL versus 6295 cells/100μL, p = 0.006) (**Figures 5A, B**). We also performed a statistical analysis for the mean of the median values of CAR T-cell percentages and CAR T-cell counts/100μL in PB following infusions of CD19m CAR-T versus CD19hs CAR-T within 30 days of enrolled patients (n=8) (CAR transgene copy number was repeatedly tested 7 times within 30 days after infusions.). As shown in **Figures S7A, B**, the means of the medians of CD19hs CAR T-cell percentages and cell counts after the 1^st^ infusions were 5.65% (range, 0.77%-10.17%) and 1201/100μL in PB (range, 90.70/100μL-3080/100μL), which were markedly higher than those after the 1^st^ infusion of CD19m CAR-T, respectively (mean of the median of percentages, 0.35% with a range of 0.0%-0.95%; mean of the median of cell counts, 133.3/100μL with a range of 0/100μL-397.8/100μL; p = 0.0036 for percentages; p = 0.049 for cell counts). The results suggest that CD19hs CAR T-cells have more proliferation activity compared to CD19m CAR T-cells.

Moreover, we observed that CD19hs CAR-T could be successfully expanded in a repeated dosing regimen. Two patients (No.4 and 5) received second infusions of CD19hs CAR-T after CR that was maintained for 11 months following the 1^st^ infusion. A second infusion dosage of 3×10^6/kg (the same as the 1^st^ dosage) was used for both patients. After treatment, we measured the CAR copy number, CAR-T percentage and cell count in blood (**Figure S8**). The copy number peak values following the second infusions were 6.04 log_10_/μg of gDNA in patient No.4 (compared to the peak value after the 1^st^ infusion of 5.8 log_10_/μg of gDNA), and 3.98 log_10_/μg of gDNA in patient No.5 (compared to the peak value after the first infusion of 6.11 log_10_/μg of gDNA). The peak values of CAR-T percentage and cell count were 4.7%, 1250 cells/100μL (versus 2.3%, 2160/100μL after the first infusion) and 33.02%, 4300/100μL (versus 2.82%, 5900/100μL after the first infusion) in patients No.4 and No.5, respectively. The values were considerably greater than those associated with CD19m CAR-T (CAR-T%, 6.36% versus1.04%; cell count, 693 cells/100μL versus 10 cells/100μL in patient No.2), indicating that CD19hs CAR-T may retain its bioactivity even after repeated dosing (**Figure 5** and **Figures S7**, **S8**). However, comparison analysis revealed no significant differences in the median of peak values and means of medians of the CAR-T percentages and cell counts following the second infusions of CD19m CAR-T (n=5) versus CD19hs CAR-T (n=2) possibly due to a small number of patients in the CD19hs CAR-T group (**Figures 5C, D**). Future studies using a larger patient cohort is warranted to address this issue.

### Clinical Response to CD19hs CAR-T Treatment

Following CD19hs CAR-T treatment, no significant GVHD was observed in any of the patients. On Day 30 following infusions, 7 of the 8 patients (87.5%) achieved CR or CRi with negative MRD. Among these 7 patients, patient No.3 had relapsed with extramedullary involvement following a CD19m CAR-T-induced CR. After infusion with CD19hs CAR-T, patient No. 3 achieved CR in BM, PB, and in the CNS, as confirmed by cerebrospinal fluid (CSF) examination. CAR-T levels in CSF were examined on Day 30 and Day 60 following infusion of CD19hsCAR-T, and the percentages of CD19hsCAR-T were 8.54% and 1.92% on Day 30 and Day 60, respectively. Among the 8 patients, only 1 patient (No.2) failed to achieve CR on Day 30. The tumor burden in patient No. 2 decreased to 14.98% on Day 15 and bounced back to 71.84% in BM on Day 30 following CD19hs CAR-T infusion. Taken together, the first infusion of CD19hs CAR-T resulted in a CR/CRi rate of 87.5% as evaluated 30 days after infusion (**Figure 2B**). For repeated therapy, two patients received the 2^nd^ infusion of CD19hs CAR-T when they relapsed approximate 1 year after the 1^st^ hsCAR-T treatment; and both patients achieved CR 30 days after the 2^nd^ treatment. Overall, CD19hs CAR-T demonstrated a robust therapeutic efficacy and mild side effects, as compared with CD19m CAR-T (**Figure 2** and **Figure S1**).

**Figure 6:**
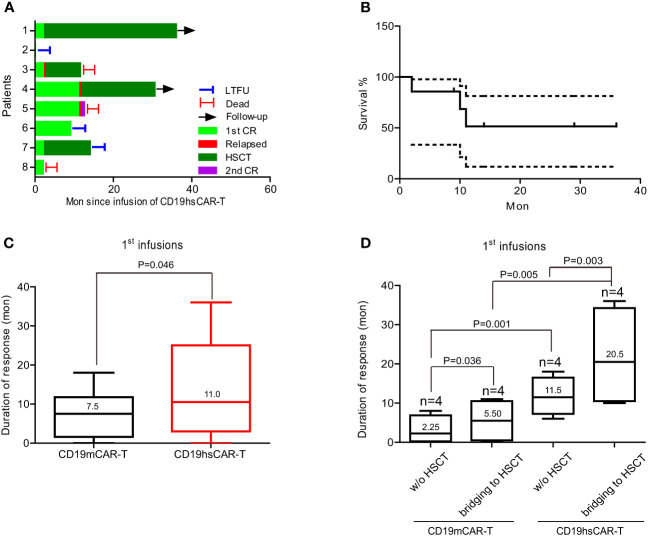
Duration of response and survival rate of patients. **(A)** The duration of response of the 8 enrolled patients after infusions. **(B)** Survival rate after infusions. **(C)** Duration of response following the 1^st^ CD19mCAR-T or CD19hsCAR-T treatments. **(D)** Breakdown of duration of response into subgroups with or without HSCT. Data are presented as median values with range. LTFU, lost to follow-up.

The median DOR was 11 months (range, 2-36 mon.) for all the patients except for patient No.2 (**Figures 6B, C**). As shown in **Figure 6A**, among these 7 patients, 2 patients (No.1 and 4) were still in CR and continued with a routine follow-up schedule as of July, 2021, when this manuscript was prepared. Patient No.1 received an allo-HSCT 2 months following the CD19hs CAR-T-induced CR. Patient No.3 received a second allo-HSCT after CD19hs CAR-T-induced CR. Nine months after a CD19hs CAR-T-induced CR, patient No. 3 died of an infection. Patients No.4 and No.5 maintained a CR with negative MRD for 11 months without supplemental treatment after the first CD19hs CAR-T infusion. Then, these 2 patients relapsed in BM and thereafter each received a second infusion of CD19hs CAR-T. Patient No.4 achieved a CR with positive MDR and then received another allo-HSCT. The patient had maintained in CMR for over 18 months when this manuscript was in preparation. Patient No.5 also achieved a CR after the second infusion of CD19hs CAR-T, but relapsed 1 month later. This patient then received a murine-based CD22 CAR-T treatment but failed to show a clinical response and died. Patient No.6 achieved CMR for 9 months without supplemental therapy following CD19hs CAR-T treatment but was lost to follow-up (LTFU) due to the COVID-19 pandemic which began in January 2020. Patient No.7 received allo-HSCT 2 months after the first CD19hs CAR-T-induced CR. This patient maintained CMR for 12 months but then was LTFU in October 2020. Patient No.8 achieved CRi with negative MDR on Day 30 following CD19hs CAR-T infusion. Two months later, however, this patient died of intracranial hemorrhage (**Tables S1**, **S6**).

Taken together, the overall response rate (ORR) with CD19hs CAR-T treatment among the 8 patients was 87.5% after the first infusion, higher than the ORR with CD19m CAR-T (62.5%). Among the patients who relapsed following a first infusion and received a second infusion, the ORR with CD19hs CAR-T and CD19m CAR-T was 100% (n=2) and 33.3% (n=3), respectively (**Figure 2B**). These clinical data demonstrate that CD19hs CAR-T results in a superior anti-tumor effect compared to CD19m CAR-T. Importantly, CD19hs CAR-T maintained anti-tumor efficacy even after repeated dosing.

### Anti-CAR Immunoglobulins in Patient Sera

To investigate the potential mechanisms underlying the primary and/or induced anti-CD19m CAR-T response, we examined the presence of specific anti-CD19m CAR immunoglobulins, including IgA, IgG and IgM, in the sera of patients before and after CD19hs CAR-T infusion. Four of the 8 patients (No. 4, 5, 6 and 8) received an HSCT following mCAR-T-induced CR and that preceded the 1^st^ hsCAR-T infusion. Since the procedure of HSCT would remove pre-existing immunoglobins in the patients, and the serum samples were not available during that specific period of time between the mCAR-T infusion and the subsequent HSCT, we divided the 8 patients into two groups in the analysis, according to whether mCAR-T was used as a bridge to HSCT (**Figure S9**). Combined with the previously published results of the first 5 patients (No.1-5) (16), and using a pre-specified cut-off-value (OD_450_>=0.2), anti-CD19m CAR IgA were positively detected in all 4 patients (No.1, 2, 3 and 7), and 1 patient serum sample (No.7) was also positive for anti-CD19m CAR IgG (**Figure S9** and **Table S4**). In the immunoglobulin-positive patients, 1 patient (No.7) exhibited primary resistance to CD19m CAR-T, and 2 patients (No.2 and 7) failed to respond to the second infusion of CD19m CAR-T. Patient No.8 exhibited primary resistance to CD19m CAR-T, and therefore received humanized CD19 CAR-T (from another hospital). This patient achieved CR and continued with a bridging to allo-HSCT regimen. Patient No.1 achieved CR after the first CD19m CAR-T infusion, but relapsed 1 month later.

We also examined anti-CD19hs CAR antibodies before and after infusions of CD19hs CAR-T. None of the patients were positive for CD19hs CAR-specific antibodies, including IgA, IgG and IgM. Patients No.4 and No.5 received the 2^nd^ infusion of CD19hs CAR-T after an 11-month CR induced by the first CD19hs CAR-T infusion. CD19hs CAR-specific antibodies were not detected before and after the second infusion of CD19hs CAR-T in any of the patients. The results suggest that CD19hs CAR probably has a lower immunogenicity than CD19m CAR, and the anti-CAR specific antibodies that were present in some patients prior to CAR-T therapy might have contributed to the primary and/or induced resistance to murine-based CD19 CAR-T.

## Discussion

CD19 CAR-T has been shown to be efficacious in treating B-cell malignancies, with a CR rate as high as 90% for B-ALL, and 60% for B-NHL, respectively, when evaluated about 1 month following infusion (1–3). However, the DOR is sub-optimal, with about 46% of treated B-ALL patients relapsed 1 year later. Additionally, 10%-20% of patients have primary resistance to murine-based CD19 CAR-T (11, 25, 26). Importantly, the overall response rate is low for those relapsing patients subjected to repeated murine-based CAR-T infusion(s), according to multiple reports (15, 16, 27). The mechanisms underlying the primary and induced resistance to mCAR-T are not fully known but may be attributed to the possible immune recognition of the murine-based scFv. In our study, patients No.5, No.7 and No.8 showed primary resistance to the first infusion of mCAR-T but achieved CR after the first humanized CAR-T infusion, implying that the difference in clinical responses might be related to the difference of scFv. Antibodies that can recognize murine-based scFv might be present in a meaningful amount in a small subset of population, and thus could neutralize the binding of mCAR with CD19 and/or suppress the proliferation/expansion of mCAR T-cells following the first CAR-T infusion. Unfortunately, serum samples from patients prior to the first mCAR-T infusion were not available from those patients who showed primary resistance, making it difficult to test for the presence of pre-existing mCAR-specific antibodies. Nevertheless, we confirmed the presence of induced anti-mCAR IgA in our study in those patients that had received CD19m CAR-T, results that are in agreement with the failed clinical response and suppressed CAR-T expansion during repeated infusion(s) of mCAR-T. *In vitro* experiments also revealed that CD19m CAR-T-mediated cytotoxicity is inhibited by the sera of patients who presented with poor clinical response and/or received multiple infusions of CD19m CAR-T without bridging to HSCT. This inhibitory effect, however, is reversed by addition of immunoglobulin-absorbing Protein-G in the co-culture system (16). In contrast, antibodies specific to hsCAR were not detected in patients before or after hsCAR-T infusions, and accordingly, repeated hsCAR-T treatments lead to a complete clinical response and marked expansion and persistence of CAR-T in some patients (**Figures S7**, **S8**), highlighting a probably lower immunogenicity and greater potency of CD19hs CAR-T compared to CD19m CAR-T.

Our study showed that CD19hs CAR-T therapy was safe, with patients exhibiting a mild CRS response (median grade of CRS, 1), even though hsCAR-T was administered at a higher dosage compared to mCAR-T (**Figure 2** and **Figure S1**). For example, Patient No. 3 relapsed with a CNS involvement and yet did not show neurotoxicity following CD19hs CAR-T treatment. The cytokine levels in sera revealed no statistical difference in hsCAR-T versus mCAR-T group following the 1^st^ or the 2^nd^ infusion(s) (**Figure 3** and **Figure S3**). However, the levels of individual cytokines showed a large variation. With a larger cohort of enrolled patients, we cannot exclude the possibility that some difference in cytokine levels may be observed. Our previous study showed that CD19hs CAR possesses a 6-fold higher affinity to CD19 than that of the mCAR counterpart [FMC63 clone (16)]. The exact reasons accounting for the better safety profile of CD19hs CAR-T are not clear, but could be due to a higher affinity, lower level of immune response to scFv, and other possible structural differences between the CAR T-cells. A well-tolerable safety profile implies that the already high dose of CD19hs CAR-T used in our study might be pushed even higher. A wider dosing range might mean that hsCAR-T may be applied to additional clinical conditions. A possible higher dosing regimen may also be conducive to removing residual cancer cells and thus achieving a MRD-negative CR status, a good prognosis indicator.

It is interesting that the DOR was significantly longer following treatment with hsCAR-T compared to mCAR-T (with or without HSCT), and longer with bridging to HSCT versus DOR without a bridging regimen (**Figure 6D**). The longer DOR with hsCAR-T may be related to a greater level of expansion and persistence in patients, possibly due to the reduced immune recognition of scFv and/or a higher proportion of a central memory T cell subpopulation in the final products, as previously reported (9, 28). The finding that a HSCT may benefit patients by extending DOR is consistent with previous reports (29–31). Most of the B-ALL patients enrolled in this study had a long disease course and treatment journey, during which, the immune system continuously was exposed to tumor cells. The relapsed/refractory state suggests that the immune system has lost its ability to fight tumor cells and a relapse implies that a number of otherwise undetectable minimal residual or newly transformed cancer cells have escaped the immune surveillance of the host and gained proliferative advantage. A failed immune surveillance in the host may be due to blunted or deficient functions of immune cells, such as the cytotoxic lymphocytes and natural killer (NK) cells that can directly kill tumor cells. Partial or complete replacement of the host immune system with one from a healthy donor through HSCT may reset the immune surveillance function and keep the cancer cell number at an undetectable level, thus delaying or even preventing relapse. The drawbacks of this approach are the enormous adverse events associated with undergoing HSCT. Since CD19hs CAR-T can be administered repeatedly without inducing immune rejection, it is reasonable to ask whether infusing hsCAR-T, particularly allogeneic hsCAR-T from identical, haploidentical, or HSCT donors, at certain intervals in a patient in a CR state could be a substitute to the bridging regimen of a HSCT. Repeated dosing of hsCAR-T as a preventive measure may help maintain a persistent presence of surveilling CAR-T cells and keep cancer cells in check. However, the optimal dosage and frequency of this approach are important to achieve relapse prevention and should be tested in future trials.

We also compared the DOR of patients without bridging to HSCT; the median was 11.5 months for CD19hs CAR-T group, which was significantly longer than CD19m CAR-T group (2.25 months, **Figure 6D**). Moreover, following the 1^st^ infusion, the percentage and cell count of hsCAR-T versus mCAR-T were higher in the peripheral blood, possibly attributed to a superior biological function of CD19hs CAR-T, such as the capacity to proliferate and/or persist (**Figure 5** and **Figures S7**, **S8**). However, given the small size of this trial, a future study with a larger cohort would be needed to verify the conclusion.

Taken together, this small phase I clinical trial demonstrated that CD19hs CAR-T is safe and efficacious in treating B-ALL patients relapsing from mCAR-T-induced CR. Importantly, CD19hs CAR-T could be repeatedly administered without the loss of efficacy. When comparing the longitudinal course of each individual patient, CD19hsCAR-T led to a significantly extended DOR compared to CD19m CAR-T therapy.

## Data Availability Statement

The datasets presented in this study can be found in online repositories. The names of the repository/repositories and accession number(s) can be found in the article/**Supplementary Material**.

## Ethics Statement

The studies involving human participants were reviewed and approved by The Ethics Committee of Xuanwu Hospital Capital Medical University, and the Ethics Committee of Hebei Yanda Lu Daopei Hospital. Written informed consent to participate in this study was provided by the participants’ legal guardian/next of kin.

## Author Contributions

Conception and design: ZC, PL, BB-S, and YZ; Data collection and assembly: YZ, JZ, JY, HW, YC, and NL; Data analysis and interpretation: YZ, ZC, HW, YC, ZL, XW, WL, and GZ; Manuscript writing: ZC, YZ, PL, and B-BS. Final approval of manuscript: All authors.

## Funding

This work was supported by the Stem Cell and Translation National Key Project (2016YFA0101403), National Natural Science Foundation of China (81973351, 82171250 and 82173840), Beijing Municipal Natural Science Foundation (5142005), Beijing Talents Foundation (2017000021223TD03), Support Project of High-level Teachers in Beijing Municipal Universities in the Period of 13th Five–year Plan (CIT & TCD20180333), Beijing Municipal Health Commission Fund (PXM2020_026283_000005), Beijing One Hundred, Thousand, and Ten Thousand Talents Fund (2018A03), and the Royal Society-Newton Advanced Fellowship (NA150482).

## Conflict of Interest

The authors declare that the research was conducted in the absence of any commercial or financial relationships that could be construed as a potential conflict of interest.

## Publisher’s Note

All claims expressed in this article are solely those of the authors and do not necessarily represent those of their affiliated organizations, or those of the publisher, the editors and the reviewers. Any product that may be evaluated in this article, or claim that may be made by its manufacturer, is not guaranteed or endorsed by the publisher.
