# Supplementary material for: Long-Term Safety and Efficacy of CD19 Humanized Selective CAR-T Therapy in B-ALL Patients Who Have Previously Received Murine-Based CD19 CAR-T Therapy
Source: Front Oncol. 2022 Jun 21;12:884782. doi: 10.3389/fonc.2022.884782 (PMC9253302; doi:10.3389/fonc.2022.884782)
Supplement: Supplementary file 1 [file DataSheet_1.pdf]

Supplementary Information

Supplementary figure legends

Figure S1. Statistical analysis of infusion dosages. (A) Comparison of the 1st and 2nd infusion dosages of CD19m CAR-T and CD19hs CAR-T. Each bar represents the dosage range with the median value as a solid straight line. (B) Distribution of infusion dosages during the 1st and 2nd treatments with CD19m CAR-T and CD19hs CAR-T.

Figure S2. Cytokine response after 1st infusions of CD19hs CAR-T and CD19m CAR-T in patients. (A) and (B) The kinetics of sCD25 after infusions of CD19hs CAR-T and CD19m CAR-T, respectively. (C) and (D) The kinetics of IL-6 after infusion of CD19hs CAR-T and CD19m CAR-T, respectively. (E) and (F) The kinetics of IL-10 after infusion of CD19hs CAR-T and CD19m CAR-T, respectively. (G) and (H) The kinetics of IFN-γ after infusions of CD19hs CAR-T and CD19m CAR-T, respectively.

Figure S3. Cytokine response after the 2nd infusions of CD19m CAR-T and CD19hs CAR-T. (A) - (D) (A, sCD25; B, IL-6; C, IL-10 and D, IFN-γ) displayed the comparison of the median concentrations of various cytokines from patients after receiving the 2nd infusions of CD19m CAR-T (n=5) or CD19hs CAR-T (n=2). The data are shown as median values with a range of concentrations of various cytokines within 30 days after infusions. Straight lines in each bar indicate the median values. P

values were calculated by using T-test. The significant level was set as 0.05. Levels of individual cytokines were repeatedly tested 7 times within 30 days after infusions.

Figure S4. CD19hs CAR-T expansion and persistence after the 1st infusion in patients. (A) CD19hs CAR gene copy numbers (n=8) after infusions. (B) The relative fold change of CD19hs CAR-T transgene copy numbers after infusions in patients. (C) Cell count of CD19hs CAR-T in PB. (D) Proportions of CD19hs CAR-T in PB after infusions. The data about Patient 1 to 5 in Figure S4 were cited from a previous publication with PMID: 31300451.

Figure S5. The proportions and cell counts of CD19m CAR-T and CD19hs CAR-T in the peripheral blood (PB) of patients No. 1 to 5. (A) and (B) The kinetics of CAR-T percentage and of CAR-T cell counts in the PB of patient No. 1. (C) and (D) The kinetics of CAR-T percentage and of CAR-T cell counts in the PB of patient No. 2. (E) and (F) The kinetics of CAR-T percentage and of CAR-T cell counts in the PB of patient No. 3. (G) and (H) The kinetics of CAR-T percentage and of CAR-T cell counts in the PB of patient No. 4. I and J, The kinetics of CAR-T percentage and of CAR-T cell counts in the PB of patient No. 5. The red dotted lines represented the 1st infusions of CD19hs CAR-T, the green solid lines represented the 1st infusions of CD19m CAR-T, the blue dotted lines represented the 2nd infusion of CD19m CAR-T. The black arrows indicated the time of the consecutive 2nd infusions of CD19m CAR-T. Figure S5 was cited from a previous publication with PMID: 31300451.

Figure S6. The proportions and cell counts of CD19m CAR-T and CD19hs CAR-T in the PB of patients No. 6 to 8. (A) and (D) The proportions and cell counts of CAR-T in PB of patient No. 6. (B) and (E) Patient No. 7. (C) and (F) Patient No. 8. The red dotted line represent the 1st infusions of CD19hs CAR-T; The green solid lines represent the infusion(s) of CD19mCAR-T. The black arrows indicate the time of the consecutive 2nd infusions of CD19m CAR-T.

Figure S7 Comparisons of the mean values of the median CAR-T percentages and cell counts in the PB within 30 days after the 1st and 2nd infusions of CD19m CAR-T and CD19hs CAR-T, respectively. (A) and (B) the mean values of median CAR-T percentages and cell counts within 30 days after the 1st infusions of CD19m CAR-T (n=8) and CD19hs CAR-T (n=8). Results are shown as scatter dot plots of the mean values with range. (C) and (D) the mean values of median CAR-T percentages and cell counts in PB within 30 days after the 2nd infusions of CD19m CAR-T (n=5) and CD19hs CAR-T (n=2). Results are shown as scatter dot plots of the means with range. P values were determined by using T-test, and the significant levels were set as 0.05. Each parameter was repeatedly tested 7 times within 30 days after infusions.

Figure S8. Expansion and persistence of CD19hs CAR-T after the 1st and 2nd infusions. (A) and (B) The copy number and relative fold change of hsCAR after the 1st and 2nd infusions of CD19hs CAR-T. (C) - (H) Comparisons of CAR-T percentage

and cell counts after the 1st and 2nd infusions of CD19m CAR-T (C and F in Patient No. 2) and CD19hs CAR-T (D and G in Patient No. 4; E and H in Patient No. 5). Figure S8C and Figure S8F were cited from a previous publication with PMID: 31300451.

Figure S9. Examination of CAR-specific antibodies in the sera of patients before and after CD19hs CAR-T infusions. (A) Anti-CAR immunoglobulins, including IgA, IgG and IgM were measured in the sera of patients who had received CD19m CAR-T without bridging to HSCT before and after CD19hsCAR-T infusions (n=4). (B) Anti-CAR immunoglobulins, including IgA, IgG and IgM were measured in the sera of patients who had received CD19m CAR-T bridging to allo-HSCT prior to CD19hs CAR-T infusions (n=4). (C) Anti-CAR antibodies were measured in serum samples from healthy donors (n=2). The cut-off-value of OD450 was set as 0.2. The data are shown as mean ± SEM by using scatter dot plots. The data about 3 of the 4 patients used in Figure S9 were cited from a previous publication with PMID: 31300451.

Figure S1

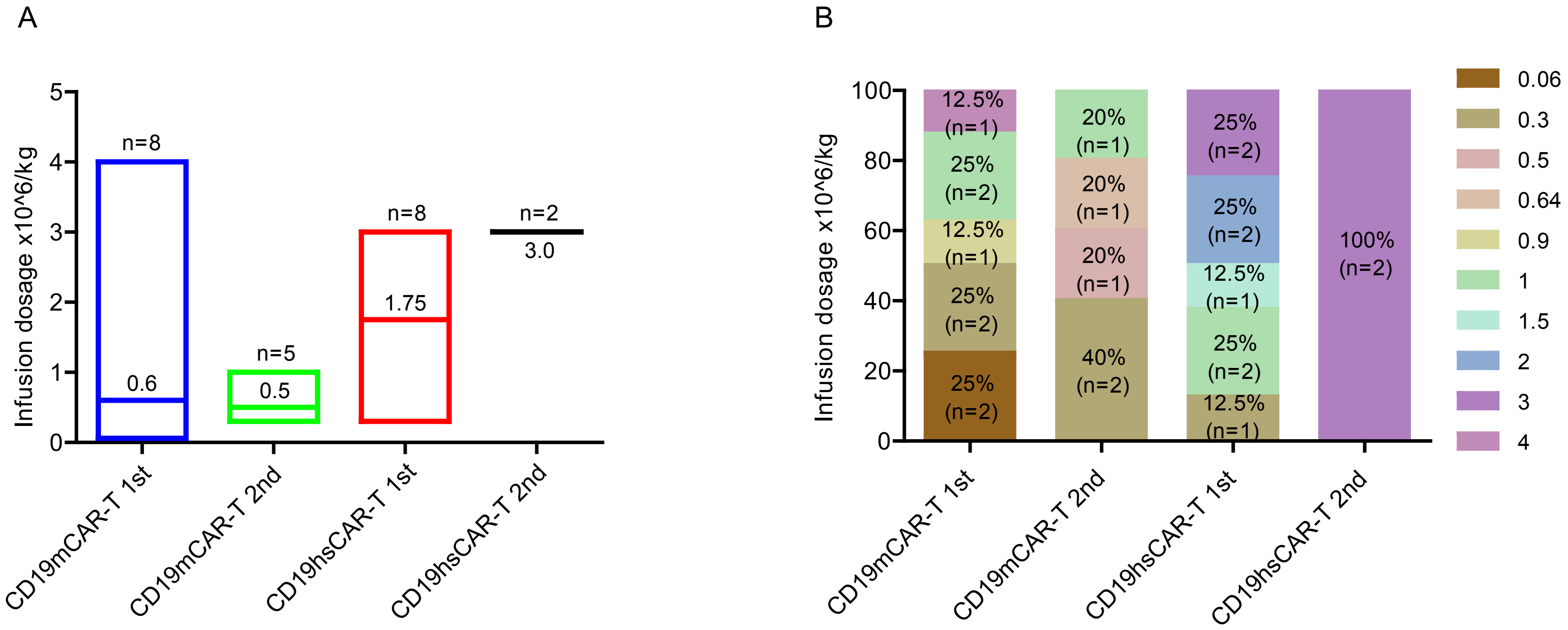
A
Infusion dosage x10^6/kg
n=8
0.6
n=5
0.5
n=8
1.75
n=2
3.0
CD19mCAR-T 1st
CD19mCAR-T 2nd
CD19hsCAR-T 1st
CD19hsCAR-T 2nd
B
Infusion dosage x10^6/kg
12.5% (n=1)
25% (n=2)
12.5% (n=1)
25% (n=2)
25% (n=2)
20% (n=1)
20% (n=1)
20% (n=1)
40% (n=2)
25% (n=2)
25% (n=2)
12.5% (n=1)
25% (n=2)
12.5% (n=1)
100% (n=2)
0.06
0.3
0.5
0.64
0.9
1
1.5
2
3
4
CD19mCAR-T 1st
CD19mCAR-T 2nd
CD19hsCAR-T 1st
CD19hsCAR-T 2nd

Figure S2

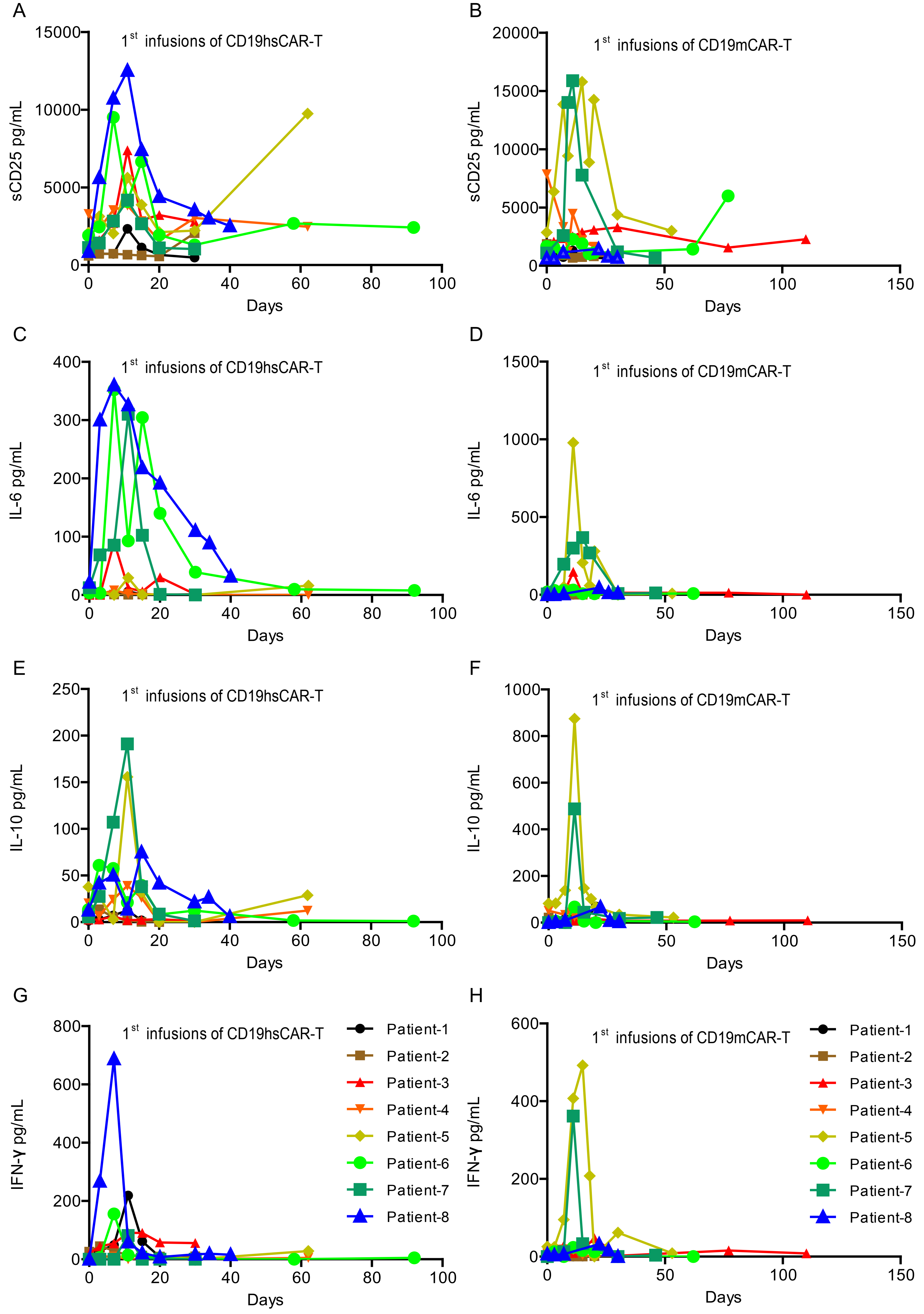
A
1st infusions of CD19hsCAR-T
sCD25 pg/mL
Days
B
1st infusions of CD19mCAR-T
sCD25 pg/mL
Days
C
1st infusions of CD19hsCAR-T
IL-6 pg/mL
Days
D
1st infusions of CD19mCAR-T
IL-6 pg/mL
Days
E
1st infusions of CD19hsCAR-T
IL-10 pg/mL
Days
F
1st infusions of CD19mCAR-T
IL-10 pg/mL
Days
G
1st infusions of CD19hsCAR-T
IFN-γ pg/mL
Days
H
1st infusions of CD19mCAR-T
IFN-γ pg/mL
Days
Patient-1
Patient-2
Patient-3
Patient-4
Patient-5
Patient-6
Patient-7
Patient-8

Figure S3

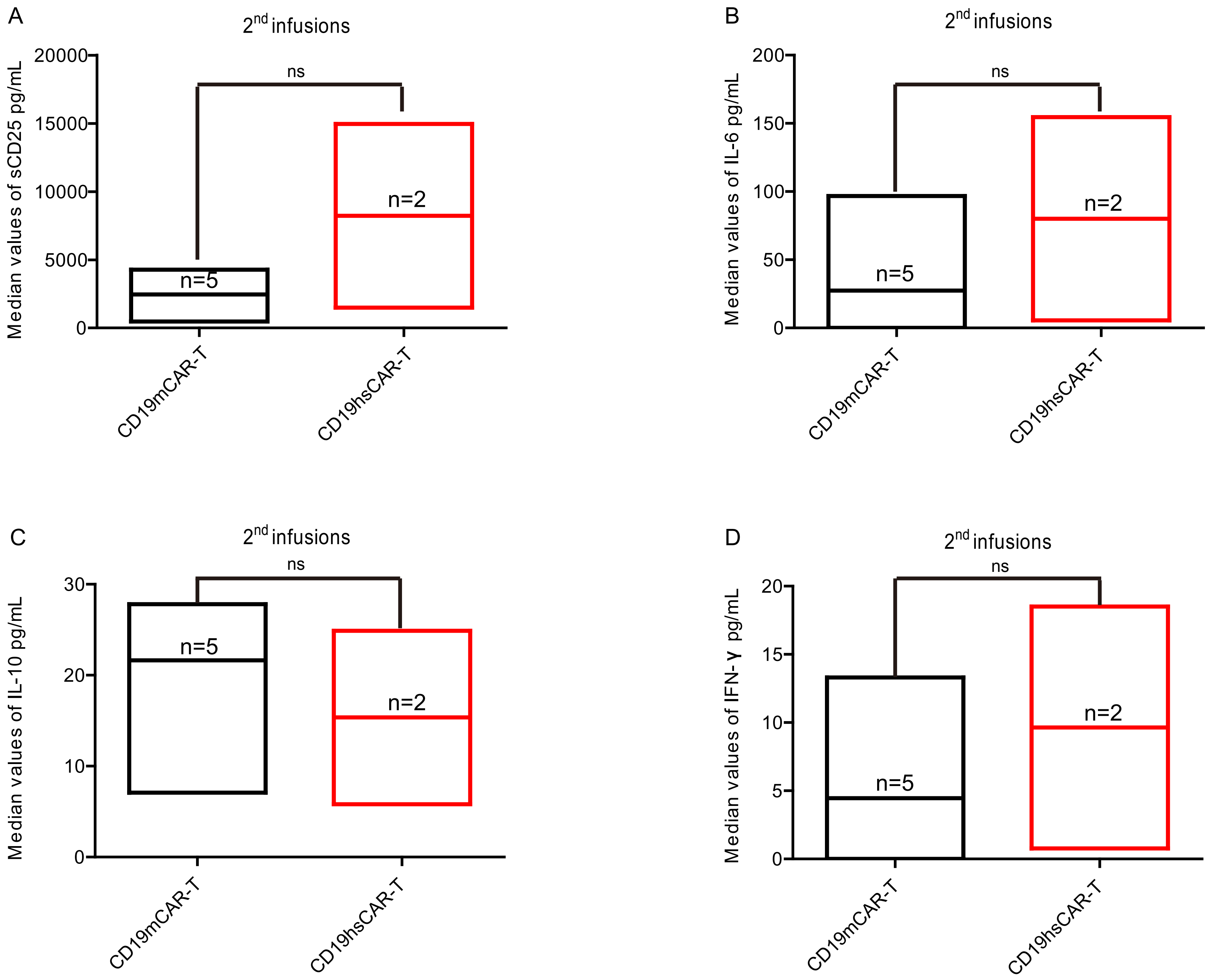
A
2nd infusions
Median values of sCD25 pg/mL
20000
15000
10000
5000
0
ns
n=5
n=2
CD19mCAR-T
CD19hsCAR-T
B
2nd infusions
Median values of IL-6 pg/mL
200
150
100
50
0
ns
n=5
n=2
CD19mCAR-T
CD19hsCAR-T
C
2nd infusions
Median values of IL-10 pg/mL
30
20
10
0
ns
n=5
n=2
CD19mCAR-T
CD19hsCAR-T
D
2nd infusions
Median values of IFN-γ pg/mL
20
15
10
5
0
ns
n=5
n=2
CD19mCAR-T
CD19hsCAR-T

Figure S4

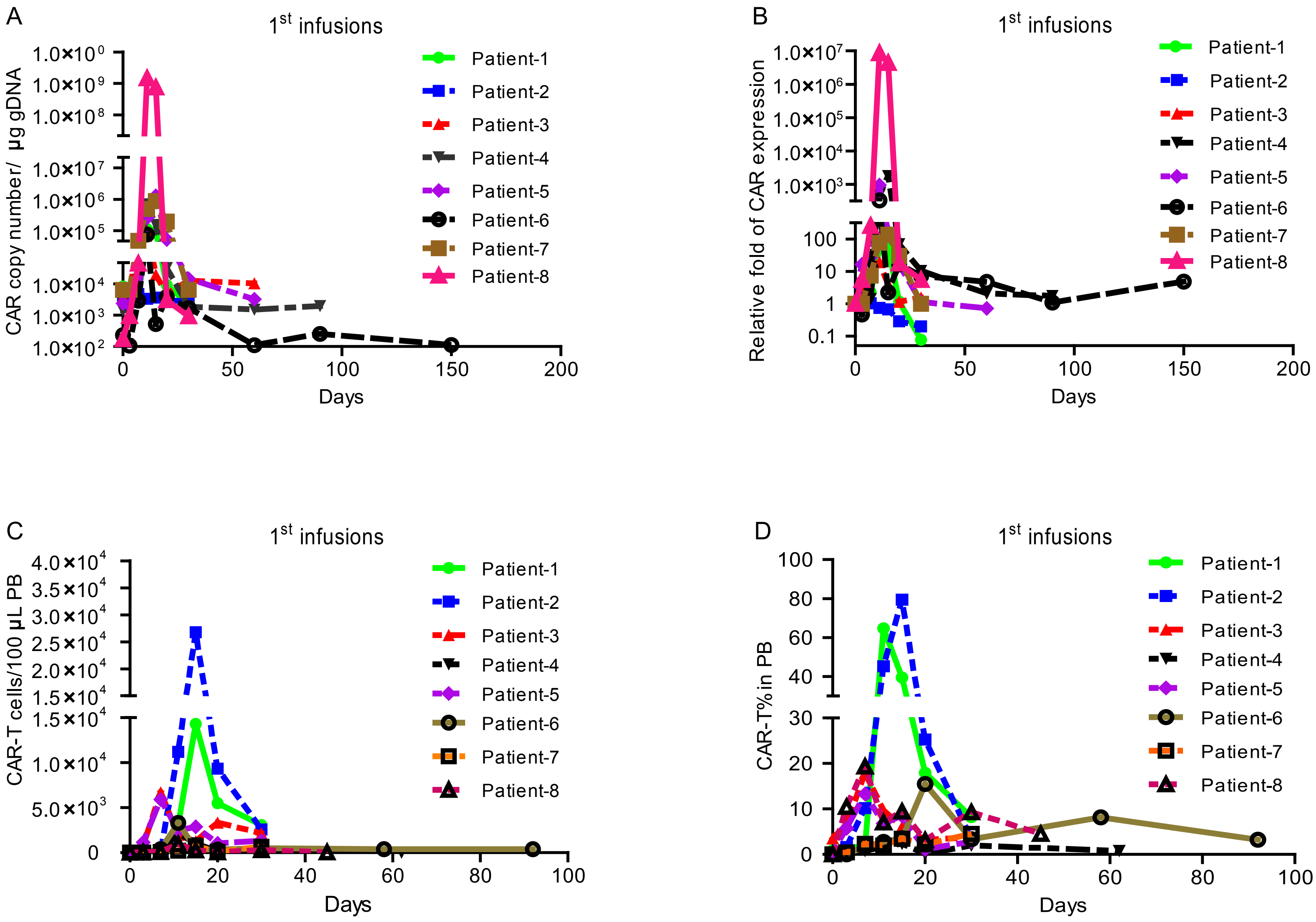
A
1st infusions
CAR copy number/ μg gDNA
Days
Patient-1
Patient-2
Patient-3
Patient-4
Patient-5
Patient-6
Patient-7
Patient-8
B
1st infusions
Relative fold of CAR expression
Days
C
1st infusions
CAR-T cells/100 μL PB
Days
D
1st infusions
CAR-T% in PB
Days

Figure S5

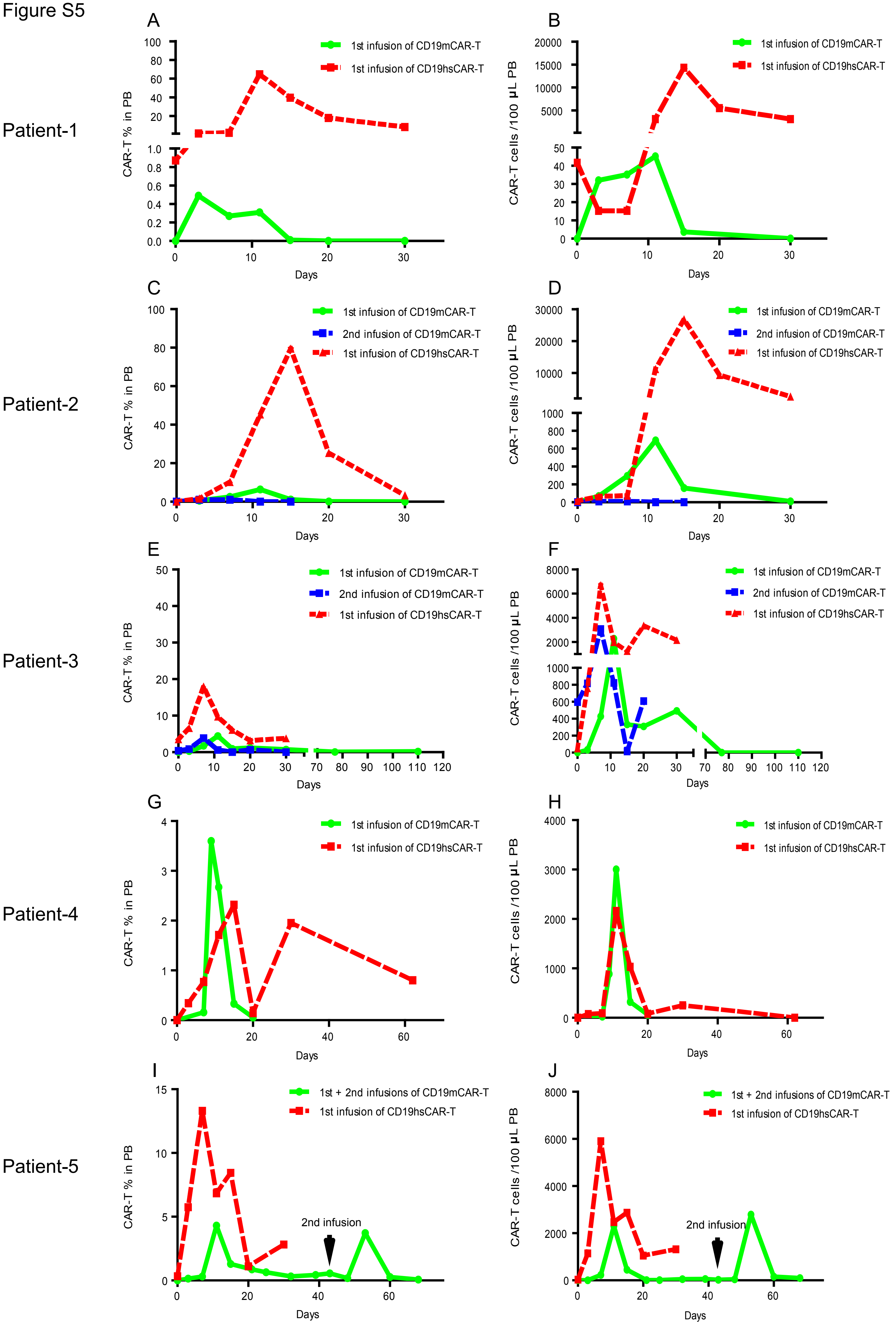

Patient-1
Patient-2
Patient-3
Patient-4
Patient-5
A
B
C
D
E
F
G
H
I
J
CAR-T % in PB
CAR-T cells /100 µL PB
Days
1st infusion of CD19mCAR-T
2nd infusion of CD19mCAR-T
1st infusion of CD19hsCAR-T
1st + 2nd infusions of CD19mCAR-T
2nd infusion

Figure S6

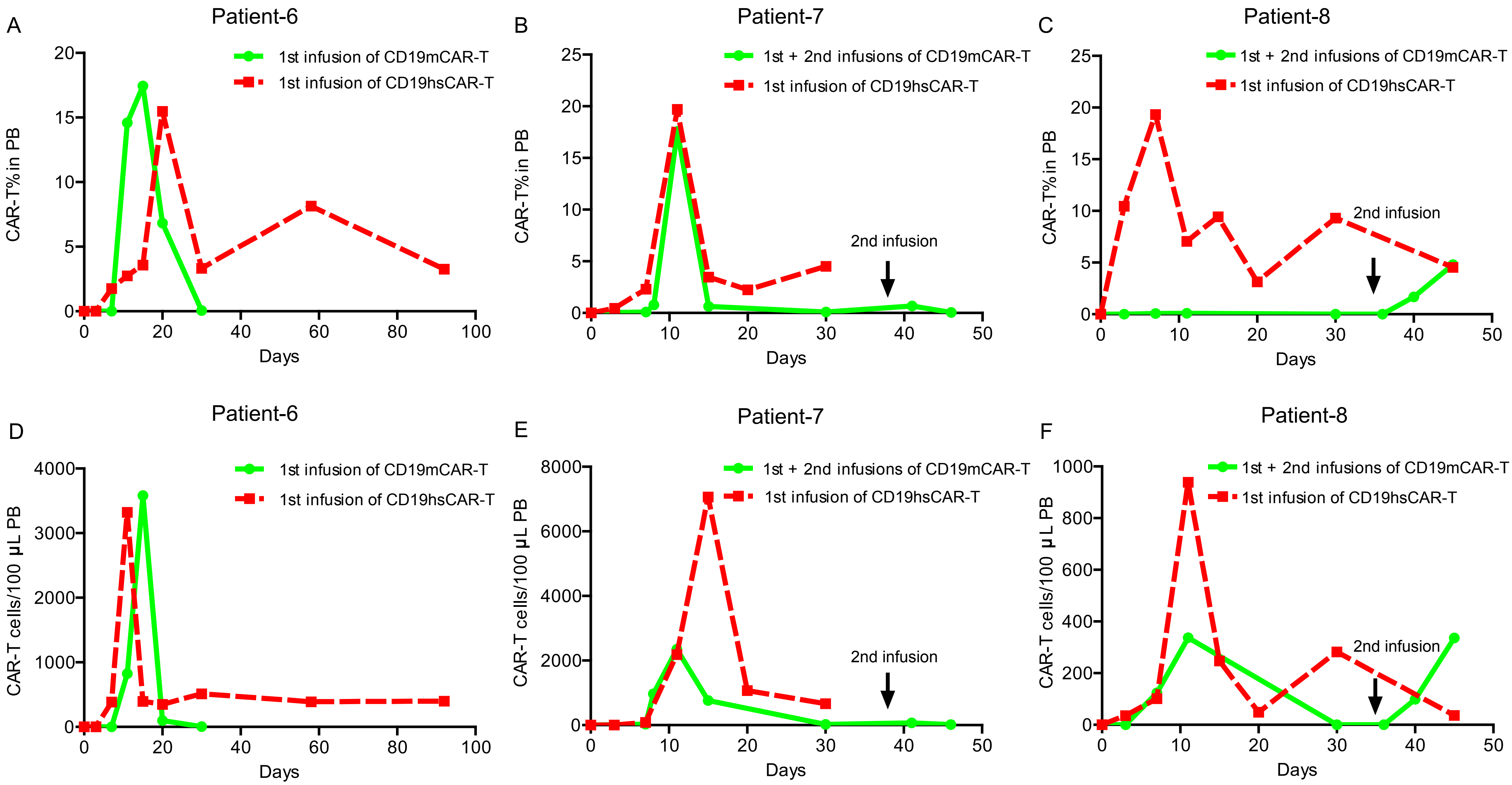
A
Patient-6
1st infusion of CD19mCAR-T
1st infusion of CD19hsCAR-T
CAR-T% in PB
Days
B
Patient-7
1st + 2nd infusions of CD19mCAR-T
1st infusion of CD19hsCAR-T
2nd infusion
CAR-T% in PB
Days
C
Patient-8
1st + 2nd infusions of CD19mCAR-T
1st infusion of CD19hsCAR-T
2nd infusion
CAR-T% in PB
Days
D
Patient-6
1st infusion of CD19mCAR-T
1st infusion of CD19hsCAR-T
CAR-T cells/100 μL PB
Days
E
Patient-7
1st + 2nd infusions of CD19mCAR-T
1st infusion of CD19hsCAR-T
2nd infusion
CAR-T cells/100 μL PB
Days
F
Patient-8
1st + 2nd infusions of CD19mCAR-T
1st infusion of CD19hsCAR-T
2nd infusion
CAR-T cells/100 μL PB
Days

Figure S7

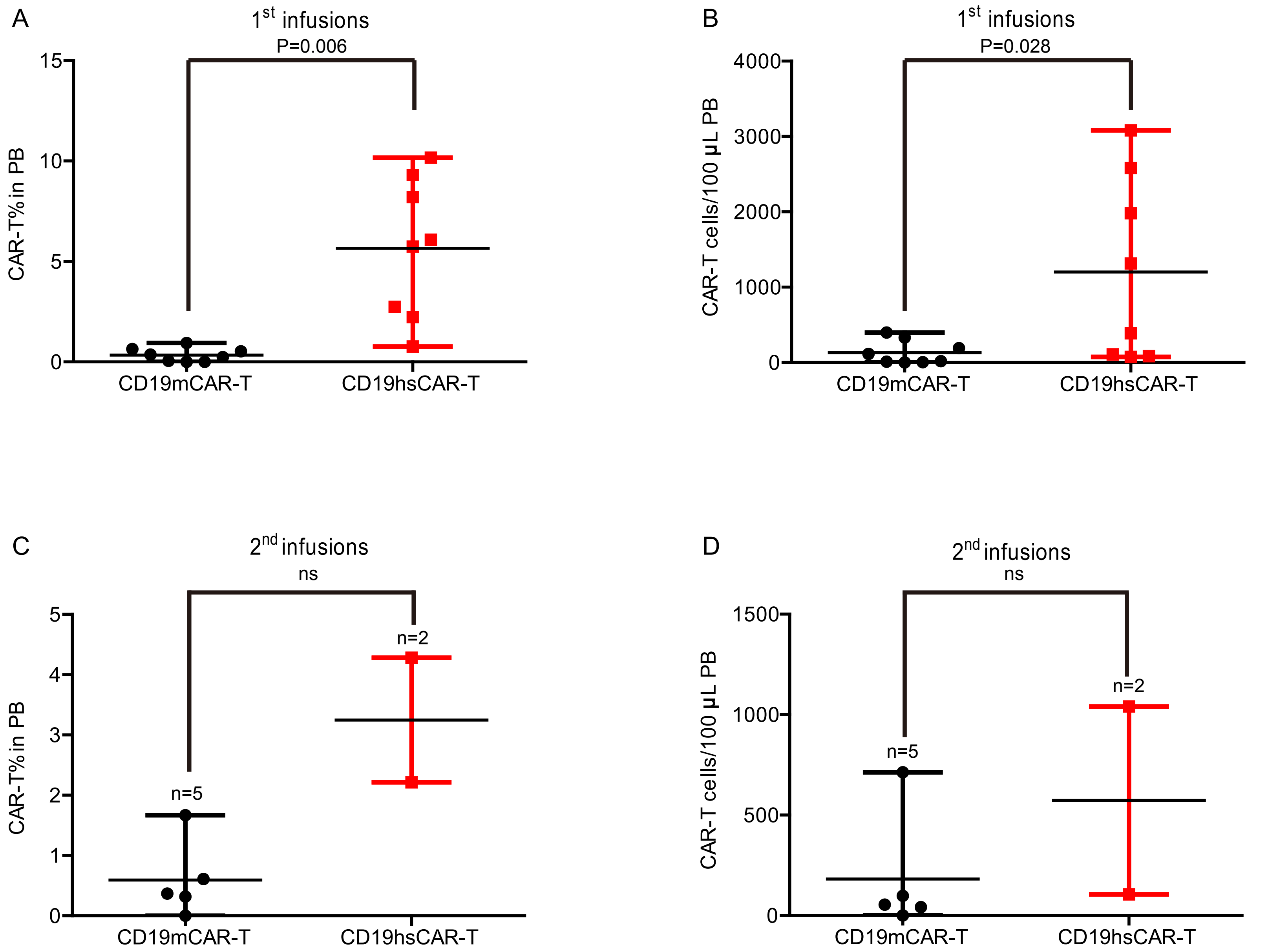

A
1st infusions
P=0.006
CAR-T% in PB
0
5
10
15
CD19mCAR-T
CD19hsCAR-T
B
1st infusions
P=0.028
CAR-T cells/100 µL PB
0
1000
2000
3000
4000
CD19mCAR-T
CD19hsCAR-T
C
2nd infusions
ns
CAR-T% in PB
0
1
2
3
4
5
n=5
n=2
CD19mCAR-T
CD19hsCAR-T
D
2nd infusions
ns
CAR-T cells/100 µL PB
0
500
1000
1500
n=5
n=2
CD19mCAR-T
CD19hsCAR-T

Figure S8

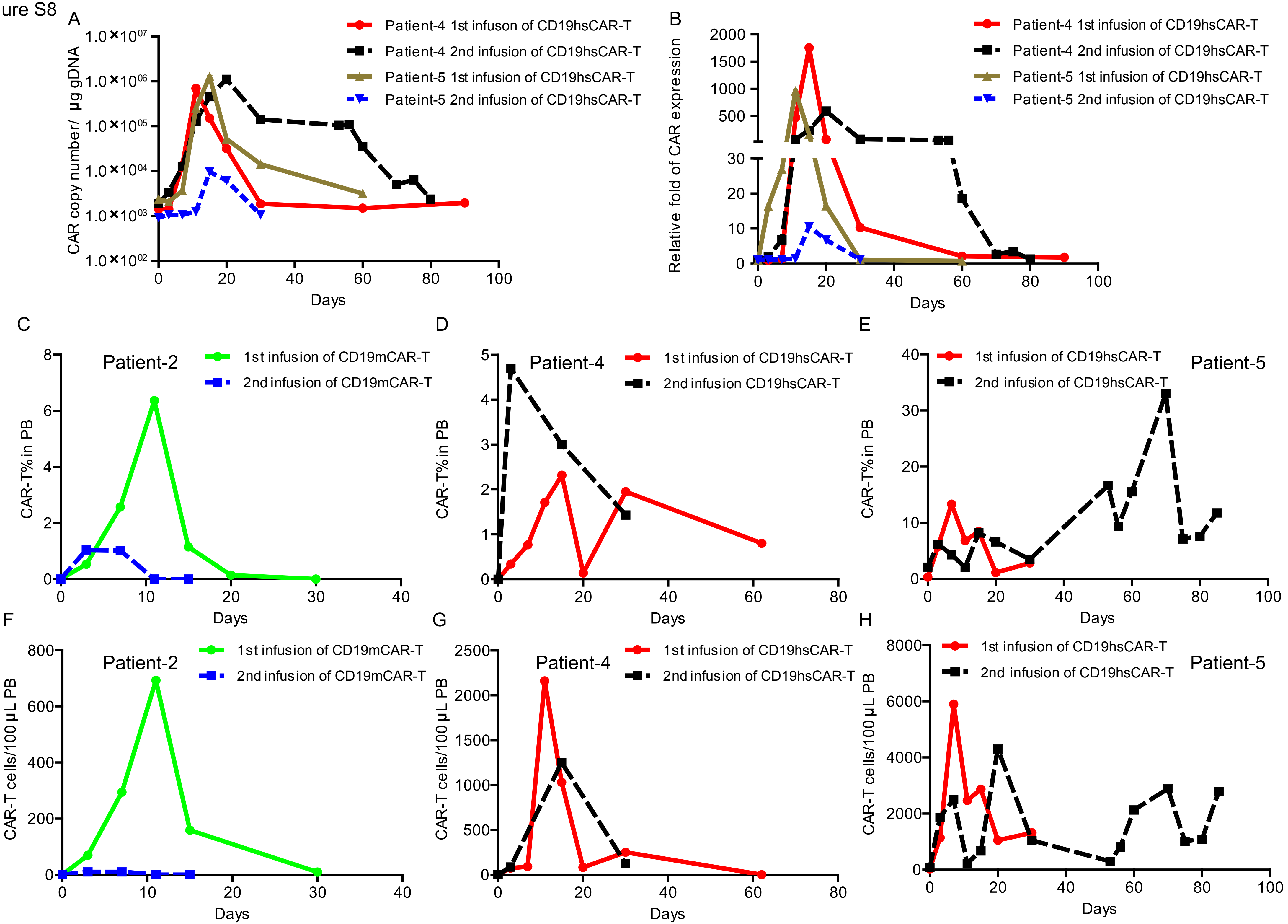

A
Patient-4 1st infuson of CD19hsCAR-T
Patient-4 2nd infusion of CD19hsCAR-T
Patient-5 1st infusion of CD19hsCAR-T
Pateint-5 2nd infusion of CD19hsCAR-T
CAR copy number/ µg gDNA
Days
B
Patient-4 1st infusion of CD19hsCAR-T
Patient-4 2nd infusion of CD19hsCAR-T
Patient-5 1st infusion of CD19hsCAR-T
Patient-5 2nd infusion of CD19hsCAR-T
Relative fold of CAR expression
Days
C
Patient-2
1st infusion of CD19mCAR-T
2nd infusion of CD19mCAR-T
CAR-T% in PB
Days
D
Patient-4
1st infusion of CD19hsCAR-T
2nd infusion CD19hsCAR-T
CAR-T% in PB
Days
E
1st infusion of CD19hsCAR-T
2nd infusion of CD19hsCAR-T
Patient-5
CAR-T% in PB
Days
F
Patient-2
1st infusion of CD19mCAR-T
2nd infusion of CD19mCAR-T
CAR-T cells/100 µL PB
Days
G
Patient-4
1st infusion of CD19hsCAR-T
2nd infusion of CD19hsCAR-T
CAR-T cells/100 µL PB
Days
H
1st infusion of CD19hsCAR-T
2nd infusion of CD19hsCAR-T
Patient-5
CAR-T cells/100 µL PB
Days

Figure S9

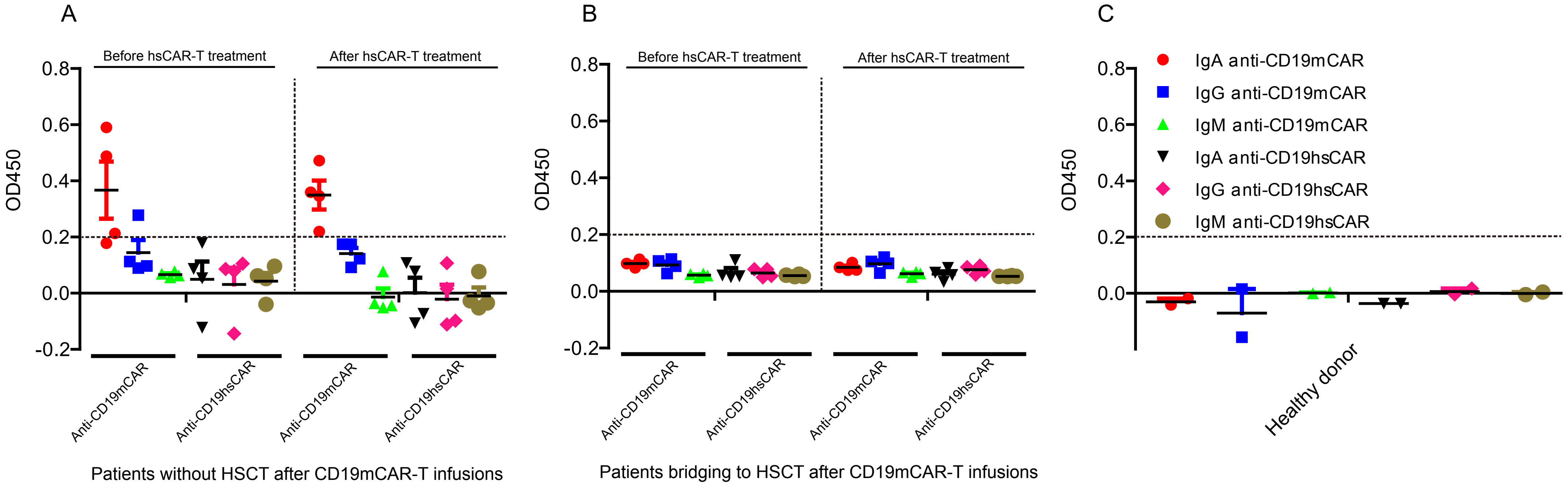

A
Before hsCAR-T treatment
After hsCAR-T treatment
OD450
0.8
0.6
0.4
0.2
0.0
-0.2
Anti-CD19mCAR
Anti-CD19hsCAR
Anti-CD19mCAR
Anti-CD19hsCAR
Patients without HSCT after CD19mCAR-T infusions
B
Before hsCAR-T treatment
After hsCAR-T treatment
OD450
0.8
0.6
0.4
0.2
0.0
-0.2
Anti-CD19mCAR
Anti-CD19hsCAR
Anti-CD19mCAR
Anti-CD19hsCAR
Patients bridging to HSCT after CD19mCAR-T infusions
C
OD450
0.8
0.6
0.4
0.2
0.0
IgA anti-CD19mCAR
IgG anti-CD19mCAR
IgM anti-CD19mCAR
IgA anti-CD19hsCAR
IgG anti-CD19hsCAR
IgM anti-CD19hsCAR
Healthy donor

Table S1. Major treatments for the enrolled patients in this study.

			Patient No.							
			1	2	3	4	5[$]	6	7	8
Major treatments and outcomes	1	Time/treatment	Sep., 2017/VDLD and CAM	Jul., 2017/FLU, CTX and VLP	Nov., 2014/VCR, DNR, L-aps, prednisone and Gleevec	Jul., 2016/Prednisone, CTX, DNR, and VDLP	Jul., 2016/CODP	Aug., 2017/CVLP and Imatinib	Mar., 2018/VDLP and VDCLP	Aug., 2017/VDLD, CAM, MTX
		Outcomes	CR with MRD+ and E2A-HLF+ for 3 mon, and then relapsed in BM with CD19+ B-ALL	CR with MRD+ and E2A-HLF+ for 5 mon, and then relapsed in BM with CD19+ B-ALL	CR with MRD+ and BCR-ABL1+ for 12 mon, and then relapsed in the CNS with CD19+ B-ALL	CR with MRD+ for 1 mon, then relapsed	Achieved CR with MRD- for 2 mon	CR with MRD+ and BCR-ABL1+ for 1 mon, then relapsed	Achieved CR with MDR+ for 3 mon	CR with MDR- for 12 mon via long-term chemotherapy, and then relapsed in BM
	2	Time/treatment	Dec., 2017/CTX Ara-C phase I and II	Nov., 2017/CD19mCAR-T (0.3×10^6/kg)	Dec., 2015/MTX, L-aps and Dasatinib combined with intrathecal chemotherapy with Ara-C, Dex and MTX	Sep., 2016/CAM, hyper-CVAD/A, L-asp and Dex	Sep.2016~ Jan., 2017/Hyper-CVAD /B ×2, VP, CVAD/A, hyper-CVAD/B for consolidation therapy	Sep. ~Oct., 2017/CAM and Imatinib	Jul., 2018/MA for consolidation therapy	Aug., 2018/HR1, HR2 and HR3
		Outcomes	NR with MRD+ and E2A-HLF+	CR with MRD- and E2A-HLF- for 3 mon, then relapsed in BM with CD19+ B-ALL	CR with MRD- and BCR-ABL1+ without extramedullary disease in the CNS for 4 mon, and then relapsed in the CNS with CD19+ B-ALL	NR with MRD+, and then relapsed in PB and BM with CD19+ B-ALL	CR with MRD- for 7 mon in total, and then relapsed in PB and BM with CD19+ B-ALL	NR with relapse and BCR-ABL1+	Maintaining CR with MDR+ for 4 mon in total	CR with MDR+
	3	Time/treatment	Feb., 2018/MTX, Dex and L-asp	Apr., 2018/CD19mCAR-T combined with CD22mCAR-T (0.3×10^6/kg for each)	Apr., 2016/IDA, Dex, and L-asp	Nov., 2016/CD19mCAR-T (1×10^6/kg)	Feb.~ May, 2017/Hyper-CVAD /A,VDLP and VLP	Nov., 2017/CTX, Dex, Dasatinib and VILP	Aug., 2018/VDCLP for consolidation therapy	Dec., 2018/Auto-CD19m CAR-T (6×10^4/kg)
		Outcomes	NR with MRD+ and E2A-HLF+	NR with tumor burden increased	CR with MRD- and BCR-ABL1- without extramedullary disease in the CNS	CR with MRD- for 1 mon	NR	NR	Maintaining CR with MDR+ for 8 mon in total, and then relapsed in BM	NR with tumor burden increased
	4	Time/treatment	Mar., 2018/FLU, IDA and CTX	May, 2018/CD19hsCAR-T (0.3×10^6/kg)	May, 2016/Haploidentical allo-HSCT (mother as donor)	Jan., 2017/Allo-HSCT followed by donor-derived NK infusions ×2 (full-match, sister as donor)	Jun., 2017/CD19mCAR-T (1×10^6/kg) $	Jan., 2018/CVLP and Ponatinib	Jan., 2019/DAC, CTX, MIT, VP-16	Jan., 2019/VNLD and humanized CD19CAR-T (6.4×10^5/kg)
		Outcomes	NR with MRD+ and E2A-HLF+	Tumor burden was decreased at Day15 after infusion, but NR at Day30 after infusion	CR with MRD- and BCR-ABL1- without extramedullary disease in the CNS for 12 mon, and then relapsed in the CNS with CD19+ B-ALL	CR for 18 mon, and then relapsed in PB and BM with CD19+ B-ALL	NR	NR with tumor burden increased	NR with tumor burden increased	CR with MDR+
	5	Time/treatment	Apr., 2018/CD19mCAR-T combined with CD22mCAR-T (0.3×10^6/kg for		Aug., 2017/CD19mCAR-T (4×10^6/kg), followed by donor-derived NK infusions x5;	Sep., 2018/CD19hsCAR-T (3×10^6/kg)	Jul., 2017/CD19mCAR-T (1×10^6/kg) $	Feb., 2018/CD19mCAR-T (0.9×10^6/kg) combined with CD22mCAR-T	Apr., 2019/VLP	Mar., 2019/Haploidentical allo-HSCT (mother as donor)

			each)		Mar., 2018/CD19mCAR-T (0.3×10^6/kg)			(1×10^6/kg)		
		Outcomes	CR with MRD- and E2A-HLF- for 1mon, and then relapsed in BM with CD19+ B-ALL		CR with MRD- and BCR-ABL1- without extramedullary disease in the CNS for 8 mon, then relapsed in BM and the CNS	CR with MDR- for 11 mon. and then relapsed with CD19+ B-ALL in BM.	CR with MRD-	CR with MRD-	Tumor burden decreased but failed to achieve CR	CR with MDR- for 6 mon, and then relapsed in BM
	6	Time/treatment	May, 2018/CD19hsCAR-T (1.0×10^6/kg)		May, 2018/CD19hsCAR-T (1×10^6/kg)	Sep., 2019/VLP, Flu and CTX	Aug., 2017/Haploidentical allo-HSCT (father as donor)	Mar., 2018/Haploidentical HSCT (father as donor)	Jun., 2019/auto-CD19mCAR-T (6×10^4/kg)	Oct. 2019/CTX, VP-16 and Bortezomib
		Outcomes	CR with MRD- and E2A-HLF- for 2 mon		CR with MRD- and BCR-ABL1- without extramedullary disease in the CNS for 2 mon, and then relapsed only in the CNS with CD19+ B-ALL	NR with tumor burden increased.	CR with MDR- for 12 mon, and then relapsed in BM with CD19+ B-ALL	CR with MRD- for 11 mon, and then relapsed in BM with CD19+ B-ALL	Tumor burden decreased, but failed to achieve CR	NR with tumor burden increased
	7	Time/treatment	Aug., 2018/Allo-HSCT		Sep., 2018/Intrathecal chemotherapy and allo-HSCT	Nov., 2019/CD19hsCAR-T (3×10^6/kg)	Oct., 2018/CD19hsCAR-T (3×10^6/kg)	Mar., 2019/CD19hsCAR-T (1.5×10^6/kg)	Jul., 2019/Auto-CD19mCAR-T (5×10^5/kg)	Dec., 2019/Allo-CD19hsCAR-T (2×10^6/kg, mother as donor)
		Outcomes	CR for 36 mon		CR for 9 mon, and then died of infections.	CR with MDR+	CR with MRD- for 11 mon, and then relapsed in BM with CD19+ B-ALL	CR with MRD- for 9 mon. and then LTFU in Jan., 2020	NR with tumor burden increased	CRi with MDR- for 2 mon, and then died of intracranial hemorrhage
	8	Time/treatment				Dec., 2019/Allo-HSCT	Oct., 2019/MXT and PC		Aug., 2019/MTX and 6-MP	
		Outcomes				CR with MDR- for 18 mon.	NR with tumor burden increased		NR with tumor burden decreased slightly	
	9	Time/treatment					Nov., 2019/CD19hsCAR-T (3×10^6/kg)		Sep., 2019/Allo-CD19hsCAR-T (sibling sister as donor, HLA full-match) (2×10^6/kg)	
		Outcomes					CR with MDR- for 1 mon., and then relapsed again		CR with MRD-	
	10	Time/treatment					Jan., 2020/CD22mCAR-T (3×10^5/kg)		Nov., 2019/Allo-HSCT (sibling sister as donor)	
		Outcomes					NR and died of B-ALL relapse		CR with MDR- for 12 mon, and then LTFU in Oct. 2020.	
Note			CR duration was calculated to the date when this manuscript was prepared.		The second infusion of CD19mCAR-T was consolidating treatment to prevent relapse but failed.	CR duration was calculated to the date when this manuscript was prepared.				

Note:

6-MP, 6-mercaptopurine

Ara-C, cytarabine;

allo-HSCT, allogeneic hematopoietic stem cell transplantation;

CAM, complementary and alternative medicine;

CF, Cyclophosphamide and Fludarabine;

CTX, Cyclophosphamide;

CODP, Cyclophosphamide, Vincristine, Daunorubicin, and Prednisone;

CVLP, Cyclophosphamide, Vincristine, L-asparaginase, and Prednisone;

DAC, Doxifluridine, Adriamycin, and Cyclophosphamide;

Dex, Dexamethasone;

DNR, Daunorubicin;

FLU, Fludarabine;

HR1, DMX, VCR, CF, CTX, Ara-C, and PEG-Asp

HR2, Methotrexate, Peasparagine, Vindesine, Dexamethasone, and Ifosfamide;

HR3, Methotrexate, Peasparagine, Vindesine, Cytarabine, and Etoposide;

Hyper-CVAD/A, Cyclophosphamide, Vincristine, Doxorubicin;

Hyper-CVAD/B, Methotrexate and Cytarabine;

IDA, Idarubicin;

L-asp, L-asparaginase;

LTFU, lost to follow-up;

MA, Mitoxantrone;

MIT, Mitoxantrone;

MTX, Methotrexate;

NR, nonresponse;

PC, Paclitaxel and Carboplatin;

PEG-Asp, PEG-asparaginase;

VCR, Vincristine;

VDLD, Vincristine, Daunorubicin or Doxorubicin, L-asparaginase, and Prednisone or Dexamethasone;

VDLP, Vincristine, Daunorubicin, L-asparaginase and Prednisone

VDCLP, Vincristine, Daunorubicin, Cyclophosphamide, L-asparaginase and Prednisone

VILP, Vincristine, Ifosfamide , L-asparaginase and Prednisone

VLP, ventriculolumbar perfusion chemotherapy;

VNLD, Vindesine, Mitoxantrone, L-Asparaginase and Dexamethasone

VP-16, Etoposide

$ Patient 5 received CD19 mCAR-T treatment in June, 2017. On day 43 post-treatment, evaluation showed that the patient did not achieve CR, and the patient received the second mCAR-T treatment on the same day (Day 43), which led to a CR.

Table S2. Toxicity survey after CD19hsCAR-T treatment.

	Grade 1	Grade 2	Grade 3	Grade 4	Note
**Adverse events**					
Cytokine release syndrome	6 (75.0%)	2 (25.0%)	0	0	N/A
Febrile neutropenia	1 (12.5%)	0	0	0	N/A
Fever	5 (62.5%)	1 (12.5%)	2 (25.0%)	0	Detailed information listed in Table S3.
**Hematological adverse events**					
Anemia	0	1 (12.5%)	3 (37.5%)	0	Patient 4: the hemoglobin level was 90.4 g/L on day 0, which was decreased to 61.9 g/L on day 47 after infusion (grade 3).<br>Patient 5: the hemoglobin level was 100 g/L on day 0, which was decreased to 81.5 g/L on day 2 after infusion (grade 2).<br>Patient 6: the hemoglobin level was 91 g/L on day 0, which decreased to 67.6 g/L on day 8 after infusion (grade 3).<br>Patient 8: the hemoglobin level was 90.3 g/L on day 0, which was decreased to 63 g/L on day 30 after infusion (grade 3).
Decreased neutrophil count	0	1 (12.5%)	0	2 (25.0%)	Patient 4 had grade 4 neutropenia with a neutrophil count of 0.2×10^9/L on day 0; on day 9 after infusion, neutrophil count was 0.10×10^9/L (grade 4).<br>Patient 5 had grade 4 neutropenia with a neutrophil count of 0.92×10^9/L on day 0; on day 36 after infusion, neutrophil count was 0 (grade 4).
Decreased platelet count	0	0	0	4 (50.0%)	Patient 4 had grade 3 thrombocytopenia with a platelet count of 35.9×10^12/L on day 0; on day 2 after infusion, platelet count was 15.9×10^12/L (grade 4).<br>Patient 5: platelet count was 120.3×10^12/L on day 0, which was decreased to 7.9×10^12/L on day 44 after infusion (grade 4).<br>Patient 6: platelet count was 238.4×10^12/L on day 0, which was decreased to 74.4×10^12/L on day 8 after infusion (grade 4).<br>Patient 8 had grade 3 thrombocytopenia with a platelet count of 21.8×10^12/L on day 0, which was decreased to 14.7×10^12/L on day 30 after infusion (grade 4).
Decreased white blood cell count	0	1 (12.5%)	0	4 (50.0%)	Patient 4 had grade 4 leukopenia with a white cell count of 0.42×10^9/L on day 0; on day 2 after infusion, white cell count was 0.56×10^9/L (grade 4).<br>Patient 5 had grade 3 leukopenia with a white cell count of 1.08×10^9/L on day 0, which was decreased to 0.39×10^9/L on day 36 after infusion (grade 4).<br>Patient 6 had grade 3 leukopenia with a white cell count of 1.09×10^9/L on day 0, which was decreased to 0.26×10^9/L on day 5 after infusion (grade 4).<br>Patient 8 had grade 4 leukopenia with a white cell count of 0.9×10^9/L on day 0, which was decreased to 0.43×10^9/L

					on day 30 after infusion (grade 4).
**Chemical laboratory test**					
Hypokalemia	2 (25.0%)	0	0	0	N/A
**Nervous system events**					
Ataxia	0	0	0	0	N/A
Dysphasia	0	0	0	0	N/A
Headache	0	0	0	0	N/A
Tremor	0	0	0	0	N/A
Ataxia	0	0	0	0	N/A

Note: N/A, not applicable.

Table S3. Survey of body temperature after CD19hsCAR-T treatment.

**Patient No.**	**Repeat dosages**	**Infusions**	**Start time with fever (days after infusion)**	**Duration (days)**	**Peak value (℃)**	**Grade**
Patient 1	1	1st	Day 7	4	39.4	2
Patient 2	1	1st	Day 2	3	38.6	1
Patient 3	1	1st	Day 5	4	40.8	3
Patient 4	2	1st	Day 8	5	38.9	1
		2nd	Day 2	3	38.5	3
Patient 5	2	1st	Day 10	1	39.0	1
		2nd	Day 3	6	39.5	2
Patient 6	1	1st	Day 5	3	40.5	3
Patient 7	1	1st	Day 7	3	38.7	1
Patient 8	1	1st	Day 3	5	38.9	1

Table S4. Anti-CAR response in patients' sera before and after hsCAR-T treatment.

	Antibody isotypes		IgA		IgG		IgM	
			OD450					
	Detection time		Before hsCAR-T	After hsCAR-T	Before hsCAR-T	After hsCAR-T	Before hsCAR-T	After hsCAR-T
**Sample ID**	**Antigen**							
**Patient 1**		**CD19 mCAR**	**0.590755**	**0.219637**	0.0895341	0.092022	0.0562419	-0.0511632
		**CD19 hsCAR**	-0.122193	-0.105871	-0.143873	-0.098112	-0.0394008	-0.026955
**Patient 2**		**CD19 mCAR**	0.178135	**0.345866**	0.0974262	0.174307	0.0627561	-0.043684
		**CD19 hsCAR**	0.18	0.107558	0.086011	-0.111115	0.0524062	-0.034588
**Patient 3**		**CD19 mCAR**	**0.212765**	**0.472216**	0.113288	0.122573	0.0689928	-0.036198
		**CD19 hsCAR**	0.053714	-0.072719	0.105453	0.016531	0.0624112	-0.0522147
**Patient 4**	**1st**	**CD19 mCAR**	0.083113	0.074954	0.0889832	0.0983526	0.0601998	0.0625049
		**CD19 hsCAR**	0.110526	0.06637	0.0775209	0.090287	0.0528421	0.0553977
	**2nd**	**CD19 mCAR**	0.068653	0.067442	0.062543	0.058995	0.058744	0.059372
		**CD19 hsCAR**	0.083062	0.067521	0.061214	0.065424	0.068849	0.069164
**Patient 5**	**1st**	**CD19 mCAR**	0.113151	0.084973	0.107124	0.105014	0.0622423	0.0666344
		**CD19 hsCAR**	0.053769	0.034032	0.0782874	0.0856885	0.0603099	0.0525453
	**2nd**	**CD19 mCAR**	0.082129	0.100587	0.065993	0.138982	0.063606	0.072683
		**CD19 hsCAR**	0.064316	0.092629	0.16576	0.096239	0.066692	0.072322
**Patient 6**		**CD19 mCAR**	0.097709	0.101585	0.063115	0.064281	0.055453	0.067605
		**CD19 hsCAR**	0.056044	0.058322	0.054451	0.058942	0.056857	0.053025
**Patient 7**		**CD19 mCAR**	**0.487719**	**0.359786**	**0.277947**	0.174973	0.07765	0.076889
		**CD19 hsCAR**	0.087341	0.077936	0.078788	0.107246	0.095948	0.077086
**Patient 8**		**CD19 mCAR**	0.097589	0.075321	0.113756	0.120719	0.049112	0.050341
		**CD19 hsCAR**	0.050731	0.083058	0.049352	0.071573	0.052006	0.052242
**HC 1**		**CD19 mCAR**	-0.042402		0.0144479		0.0020803	
		**CD19 hsCAR**	-0.037279		0.0160554		0.0035765	
**HC 2**		**CD19 mCAR**	-0.019394		-0.1572369		-0.0015892	
		**CD19 hsCAR**	-0.037279		-0.0048031		-0.0053109	

Note:

1. Patients 1 to 8 received murine-based CD19CAR-T treatment at least once prior to CD19hsCAR-T therapy;

2. Patient 4, Patient 5, and Patient 6 received CD19hsCAR-T treatment after HSC transplantation;

3. Patient 5 displayed primary resistance to murine-based CD19CAR-T treatment, but achieved CR after the 2nd infusion;

4. Patient 7 displayed primary resistance to murine-based CD19CAR-T treatment;

5. Patient 8 displayed primary resistance to murine-based CD19CAR-T treatment, but achieved CR for 6 mon after infusion of humanized CD19CAR-T (from a different group) bridging to HSC transplantation;

6. HC 1 and HC 2 were healthy donors used as negative controls;

7. The cut-off-value for positivity was set as 0.2, and positive readings were highlighted as bold; the readings close to the cut-off-value were also highlighted in bold with dark grey background.

Table S5. Subpopulation analysis of the final products for the 8 patients.

		Patient No.							
		1	2	3	4	5	6	7	8
Starting PBMCs	CD3+ in PBMCs	29.8%	32%	6.9%	46.2%	8.1%	29.6%	8.0%	10.0%
	CD19+ in PBMCs	0.7%	0.8%	1.95%	1.0%	2.1%	4.01%	1.7%	2.1%
	CD27+CD45RO-PD-1- in CD8+	25.9%	31.4%	8.28%	38.1%	25.9%	14.3%	32.9%	14.9%
	Tcm% in CD8+/CD3+	20.6%	14.7%	29.4%	4.22%	2.93%	14.2%	OOL	0.72%
	Tem% in CD8+/CD3+	44.6%	44.9%	32.5%	38.2%	32.9%	51.9%	0.89%	15.5%
	Starting PBMC（×10^8）	2.7	2.88	3.45	2.51	2.1	3.58	2.16	3.12
	Ratio of CD4/CD8	0.54	0.68	2.3	1.87	1.35	1.77	0.77	0.64
Final Product (FP)	CD3+ in FP	98.4%	97.7%	95.0%	98.7%	91.3%	97.2%	98.0%	95.4%
	CD19+ in FP	OOL	OOL	OOL	OOL	OOL	OOL	OOL	OOL
	CD27+CD45RO-PD-1- in CD8+	12.7%	7.28%	13.75%	15.3%	48.1%	10.4%	45.1%	30.7%
	Tcm% in CD8+/CAR+	70.1%	64.9%	64.3%	61.2%	53.3%	9.47%	11.0%	61.1%
	Tem% in CD8+/CAR+	25.9%	23.5%	25.1%	16.4%	40.4%	20.6%	19.1%	27.1%
	Final product（×10^7）	57.5	58.5	67.6	31.4	35.2	76.5	53.9	68.4
	CAR% in CD3+	49.5%	39.3%	38.1%	35.1%	33.3%	36.6%	50.0%	42.3%
	Ratio of CD4/CD8	0.17	8.4	1.06	0.87	1.00	0.99	0.54	0.89

Note: FP, final product; OOL, out of limit.

Table S6. Patients' clinical responses after CD19hs CAR-T treatment

Patient No.	Before CD19hs CAR-T infusions: Tumor burden in BM: Morphology %	Before: Tumor burden in BM: Flow cytometry %	Before: CSF %	Before: Pre-B in PB %	Infusions of CD19hs CAR-T: Cell resource	HLA matching status	Repeat infusions	Dosage $\times 10^6$/kg	CRS: Grade	CRS: Neurotoxicity	CRS: Tocili	CRS: Steroid	After CD19hs CAR-T infusions: Day 15: Tumor burden in BM: Morphology %	Day 15: Tumor burden in BM: Flow cytometry %	Day 15: CSF %	Day 15: Pre-B in PB %	Day 30: Tumor burden in BM: Morphology %	Day 30: Tumor burden in BM: Flow cytometry %	Day 30: CSF %	Day 30: Pre-B in PB %	Response after 1 month	Bridging to HSCT after infusions	Follow-up
1	4	2.28	0	0	Auto	N/A	1	1	1	N	N	Y	0	0	0	0	0	0	0	0	CR with MRD-	Y	Allo-HSCT 2 mon later; CMR for 36 mon.
2	46	34.86	0	0	Auto	N/A	1	0.3	1	N	N	Y	10.5	14.98	0	0	82	71.84	0	0	NR	N/A	LTFU
3	0.02	0	66.13	0	Allo	5/10	1	1	1	N	N	Y	0	0	0	0	0	0	0	0	CR with MRD-	Y	Allo-HSCT 4 mon later; CMR for 9 mon; and then died of infection.
4	29	15.13	0	0	Allo	5/10	2	3	1	N	N	Y	0	0	0	0	0	0	0	0	CR with MRD-	N	1. CMR for 11 mon after the 1st infusion; and then relapsed in BM; 2. Achieved CR with MDR+ after the 2nd infusion bridging to allo-HSCT; CMR for 18 mon.
	NR	4.17	0	0				3	1	N	N	Y	ND	0.78	0	0	ND	0.00032	0	0	CR with MRD+	Y	
5	6	34.74	0	0	Allo	5/10	2	3	1	N	N	Y	0	0	0	0	0	0	0	0	CR with MRD-	N	1. CMR for 11 mon after the 1st infusion; and then relapsed in BM. 2. CMR for 1 mon after the 2nd infusion, and then relapsed in BM; 3. Received CD22mCAR-T, 5×10^5/kg, with nonresponse; then died of relapse.
	8	74.5	0	0				3	2	N	Y	Y	ND	9.34	0	0	ND	0	0	0	CR with MRD-	N	
6	17	5.58	0	0	Allo	5/10	1	1.5	2	N	Y	Y	0	0	0	0	0	0	0	0	CR with MRD-	N	CMR for 9 mon and then LTFU.
7	85.5	74.47	0	0	Allo	10/10	1	2	1	N	N	Y	0	0.0045	0	0	0	0	0	0	CR with MRD-	Y	Allo-HSCT 2 mon later; and maintained in CMR for 12 mon, and then LTFU in Oct. 2020.
8	25.5	33.59	0	0	Allo	5/10	1	2	2	N	N	Y	0	0	0	0	0	0	0	0	CR with MRD-	N	CRi with MRD- for 2 mon, and then died of intracranial hemorrhage.

Note:

Allo, allogeneic;

allo-HSCT, allogeneic hematopoietic stem cell transplantation;

Auto, autologous;

BM, bone marrow;

CD19hsCAR-T, chimeric antigen receptor T cells engineered with humanized selective CD19-specific scFv;

CMR, complete molecular remission;

CRS, cytokine release syndrome;

CSF, Cerebrospinal fluid;

LTFU, lost to follow-up;

MRD, minimal residual disease;

N, no;

NR, nonresponse

PB, peripheral blood;

Tocili, tocilizumab;

Y, yes.
